# On the Ovum of Man and the Mammifera before and after Fecundation

**Published:** 1843-10

**Authors:** T. Wharton Jones

**Affiliations:** Lecturer on Anatomy, Physiology, and Pathology at the Charing-Cross Hospital; Corresponding Member of the Imperial and Royal Society of Physicians and Surgeons of Vienna, &c. &c.


PART THIRD.

### Original Reports and Memoirs.

### REPORT ON THE OVUM OF MAN AND THE MAMMIFERA, BEFORE AND AFTER FECUNDATION.

By T. Wharton Jones, F.R.S.
Lecturer on Anatomy, Physiology, and Pathology at the Charing-Cross Hospital; Corresponding Member of the Imperial and Royal Society of Physicians and Surgeons of Vienna, &c. &c.*

Inasmuch as without a knowledge of the ovum before fecundation it is not possible to trace the first stages in its subsequent development, so the discovery of the ovum of the mammifera in the ovary must be viewed as the first great step towards the elucidation of generation and development in that class of animals. But as on its first discovery, the exact nature of the ovum in the ovary was mistaken, the subsequent detection in it of a germinal vesicle, by establishing its real import, must be viewed as the second great step towards the same important end. As a consequence, therefore, of these brilliant discoveries, the early development of the ova of the mammifera has now been worked out in as complete a manner as was previously the case only with the ova of birds.

### I. Ova of man and the mammifera generally, before fecundation.

§ 1. *Graafian follicles.* The Graafian follicles, formerly supposed to be themselves the ovarian ova, were proved by the discovery of the real ova to be merely their containing capsules, capsules analogous to those which contain the ovarian ovum of the bird. The wall of the Graafian follicle is composed of interwoven fibres of cellular tissue and of blood-vessels. Its inner surface, where the capillary network is expanded, is smooth, and lined by a layer of nucleated cells, which, when the follicle is laid open, escapes in the form of membranous shreds. Originally the wall of the Graafian follicle is a simple structureless membrane, (*ovisac* of Barry, *membrana propria* of Henle and Bischoff,) around which are developed the cellular tissue and vessels which constitute its thickness subsequently.

§ 2. *Contents of the Graafian follicles.* The membraniform layer of nucleated

* This Report is founded on the following very excellent works of Professor Bischoff of Heidelberg:

1. Entwickelungsgeschichte der Säugethiere und des Menschen. Von Th. L. W. Bischoff.

History of the Development of the Mammifera and Man. By Th. L. W. Bischoff. Being Vol. Seventh of the new edition of Soemmerring's Anatomy.—Leipsic, 1842. 8vo.

2. Entwickelungsgeschichte des Kaninchen-Eies. Von Th. Ludw. Wilh. Bischoff, Doctor der Medicin und Philosophie, ausserordentlichem Professor der Medicin an der Universität Heidelberg, u. s. f. Gekrönte Preisschrift, ausgesetzt von der physikalisch-mathematischen Klasse der königlich Preussischen Akademie der Wissenchaften im Jahre 1840. Mit sechszehn Steintafeln.—Braunschweig, 1842.

History of the Development of the Ovum of the Rabbit. By Th. L. W. Bischoff, M. et P.D. Extraordinary Professor of Medicine in the University of Heidelberg, &c. Being a successful Prize Essay on the question proposed by the Physical and Mathematical Class of the Royal Prussian Academy of Sciences in the year 1840. With Sixteeen Lithographic Plates.—Brunswick, 1842. 4to.

Except when otherwise stated, it is to this second work that reference is always made.

cells* lining the Graafian follicle was named *membrana granulosa* by Baer. At the place corresponding to that side of the Graafian follicle which is prominent at the surface of the ovary, the membrana granulosa is thicker, presenting a greater accumulation of cells than in the rest of its extent. This accumulation of cells was, from a false analogy with the bird's egg, called by Baer, "discus proligerus." In the central part of it, which he called "cumulus," the ovum is imbedded, and may be seen as a minute opaque white speck, shining through when the wall of the Graafian follicle is transparent.

§ 3. *Dr. Barry's alleged tunica granulosa and retinacula.* The cells immediately surrounding the ovum have been viewed by Dr. Martin Barry† as constituting a distinct layer, which he names *tunica granulosa* of the ovum. Bischoff (p. 3) objects to this, remarking that the layer of cells in question is not distinctly defined, nor is it developed separately from the rest of the cumulus of cells in which the ovum is imbedded. As to the structure or structures consisting of a central mass containing the ovum, and of cords or bands extending from it to the membrana granulosa, which Dr. Barry describes under the name of *retinacula*, and which he alleges serve first to suspend the ovum in the fluid of the Graafian follicle; next to convey it to a certain part of the periphery of this follicle; subsequently to retain it in the latter situation; and finally to promote its expulsion from the ovary—Bischoff says he has never been able to see anything of the kind. He thinks Barry has mistaken irregular processes of the membrana granulosa in a lacerated state for a natural and definite structure.

§ 4. *Ovum.* The granular mass which the ovum presents in its centre is recognized by all observers, (including Dr. Barry in his 1st and 2d series, but excepting him in his 3d series,) as analogous to the yelk of the bird's egg.‡ Respecting the broad transparent ring or *zona pellucida* at its circumference there has been less unanimity of opinion; now, however, it is pretty generally acknowledged to be the optical expression of the circumferential doubling of a thick transparent membrane which incloses the yelk.

§ 5. *Yelk.* The yelk is composed of larger and smaller granules and globules, held together by a clear viscid substance, so closely and firmly in some cases that the yelk may be extracted entire in the form of a ball from its envelope, so loosely, on the contrary, in other cases, that when the external envelope is torn, it escapes in a formless flake. The smaller granules, Henle well remarks, are the most numerous, and resemble pigment molecules both in their appearance and in their peculiar motions, the larger, about 1-5000th of an inch in diameter, resemble fat or milk globules in their rounded form, dark edges, and shining surface.

§ 6. In the appearance of the yelk there is considerable difference in different animals and at different stages of development. The more mature the yelk the denser, opaquer and more coherent it is; the more immature, the more transparent and less coherent. According to Barry, (2d series, p. 349,) the difference between the mature and immature ovum consists in the yelk of the former containing no oil-like globules (vesicles), which that of the latter does. In the mature ovum, the yelk presents a peripheral stratum, sometimes appearing granulous, and at others seeming to consist of vesicles pressed together into a polyhedral form, its centre being fluid. In his 3d series, Dr. Barry substitutes for the name of "yelk" the expression "substance by which the germinal vesicle is surrounded," and points out (§ 337, et seq.) what he believes to be "the order of these different appearances, as well as the process to which they are referrible."

* The cells measure 1-2000th of an inch in diameter, but besides them, bodies four times larger are occasionally observed though in small numbers. In these larger bodies, Bischoff believes that he has distinguished a cell-membrane and nucleus, and throws out the conjecture that they may be intended for the formation of new follicles and ova.

† Researches in Embryology, 1st series. In Philosophical Transactions, Part ii. for 1838.

‡ Reichert distinguishes in the yelk of the bird's egg the central matter under the name of *formative vitellus,* the rest under the name of *nutrient vitellus.*

§ 7. *Zona pellucida. External membrane of the ovum.* That the zona pellucida is the optical expression of the circumferential doubling of a thick transparent membrane inclosing the yelk appears to have been first stated, independently of each other, by Coste and the author of this report. Bischoff maintains this view, but has fallen into error when he attributes it to Baer, who, on the contrary, describes the appearance of zona pellucida as owing to a transparent interval between a thin external membrane and the yelk. Valentin followed Baer in this view, and supposed the transparent interval was filled with fluid. Wagner formerly entertained a similar opinion, but he now admits the matter to be as above stated.

§ 8. Could any doubt remain on the point, the following *experimentum crucis*, which the author of this report made when he first investigated the subject, must remove it: Tear open the ovum under the simple microscope, and spread out flat a portion of the external envelope, then by delicate manipulation with fine needles double the membrane on itself, when the appearance of the double contour and broad transparent interval of the zona pellucida will be produced.

§ 9. Some observations published by Krause in Müller's Archiv for 1837, introduced great confusion into the discussions on the point in question. The albuminous mass around the ovum, which he describes as zona pellucida, is not what other authors describe as such, but must have been a part of the "discus proligerus," which in some cases is transparent immediately around the ovum. Dr. Barry has added to the confusion by attesting in his 1st series to the accuracy of Krause's statement, though what he says is actually an attestation to the opposite effect. In his 2d series, (Phil. Trans. Part ii. for 1839, p. 342,) Dr. Barry retracts this attestation.

§ 10. The thick transparent external membrane which, by being seen doubled on itself at the circumference of the ovum, gives rise to the appearance called "zona pellucida,"—is it analogous to the *vitellary membrane* of the bird's egg, or is there, in addition to it, another membrane entitled to the name of *vitellary?* The great importance of this question in reference to the tracing of the future development of the ovum was a few years ago forcibly insisted on by the author of this report, when he combated what will be shown to have been erroneous views promulgated by Dr. Martin Barry on the subject. Bischoff insists on the same point and adopts the same view as the author of this report.

§ 11. Before attempting to answer the question just proposed, let it first be inquired, what constitutes a vitellary membrane: The yelk of the bird's egg is immediately surrounded by a delicate, transparent, homogeneous, structureless membrane. It was to this that the name of vitellary membrane was originally given, and any part of the mammiferous ovum to be entitled to a similar appellation must be analogous to it in all its essential relations. That the thick transparent external membrane of the mammiferous ovum is the part analogous to the vitellary membrane of the bird's egg is maintained by Coste, the author of this report, Henle, Bischoff, &c., and, notwithstanding what has been said to the contrary by Valentin, Wagner, Barry, Meyer, and others, there is certainly no other membrane of the mammiferous ovum besides it to which the name of *vitellary* can be given.

§ 12. It has been above shown that Bischoff is inaccurate in saying that Baer described correctly the thick external membrane of the ovum; it must be here added that he is equally inaccurate in what he says regarding Baer's views of a vitellary membrane. Baer, in fact, considered the outer membrane of the ovum as analogous to the membrane of the shell in the bird's egg, and he accounts for a vitellary membrane by conjecturing that there forms on the outer surface of the yelk-ball a thin pellicle. But, as has been observed by the author of this report, "there is no such pellicle as that spoken of by Baer; and all analogy leads to the supposition that the external envelope of the ovum of the mammifera does not correspond with the membrane of the shell of the bird's egg, but rather with the vitellary membrane."

§ 13. The preceding quotation and a reference to papers in vols. xxi* and xxiv† of the Medical Gazette, will show that Bischoff is mistaken in classing the author of this report with those who admit a membrane in the mammiferous ovum, in addition to the thick external one as entitled to the name of vitellary. So far indeed from admitting any such membrane himself he criticised Barry in a very decided manner for doing so. (Med. Gaz. vol. xxiv., p. 592.) The author of this report, in fact, believes he was the first to maintain that the thick external membrane of the mammiferous ovum, is analogous to the vitellary membrane of the bird's egg in all its essential relations, before as well as after‡ further development.

§ 14. The inaccurate observations of Krause regarding the zona pellucida have been just referred to. What he describes and delineates as vitellary membrane, is in fact what in this report is maintained to be not only vitellary membrane, but also the membrane which, by being seen doubled on itself at the circumference of the ovum, gives rise to the appearance called *zona pellucida*, whereas, as has been seen, Krause attributes the latter to a different cause.

§ 15. Wagner, in his Physiology, rather infers the existence of a vitellary membrane distinct from that which yields the appearance of *zona pellucida* than says he has actually observed such. The ground of his inference is that in some cases the granules and cells composing the yelk hold together independently of the external membrane. This however, it has been above seen, is owing to a uniting viscid substance, and not to any proper inclosing membrane.

§ 16. Barry does not, like Wagner, infer merely that a vitellary membrane distinct from the thick transparent external membrane of the ovum exists, but says he has seen, nay actually delineates such. In his first series he says it is not always to be found. In his third series, (Phil. Trans., Part ii, for 1840. p. 534,) he says that several such membranes successively arise and disappear in a single ovum; a circumstance, he remarks, which will perhaps serve to explain why some observers have never seen such a membrane under the thick transparent membrane. Further, according to Dr. Barry, there are formed at the periphery of the yelk in ripe ova successive layers of discs or cells, one layer liquefying, being replaced by another. It is around these successive layers, that his successive vitellary membranes are formed.

§ 17. Did such membranes really exist, they could not be admitted as analogous in all essential relations to the vitellary membrane of the bird's egg, and therefore not entitled to the name of vitellary in the conventional sense. But in regard to Barry's statements, Bischoff observes, p. 10: "Never before, nor yet since, have I, in the course of my most careful examinations of the ovarian ovum with very good instruments, (and such I possess, one by Schieck and one by Oberhäuser,) been able to perceive any trace of such a cortical layer of the yelk. . . . . . . . . It ought also to be observed that not one of the many careful observers of the ovarian ovum has seen anything of the kind. . . . . . . "But," Bischoff continues p. 11, "if I have not been able to see this cortical layer of cells, much less have I ever been able to see a fine membrane investing it."

§ 18. As to Dr. Meyer, he alleges that liquor potassæ dissolves the membrane on which the appearance of zona pellucida depends, but leaves a membrane investing the yelk. On repeating this experiment, however, Bischoff found that liquor potassæ *does not dissolve the zona*, but merely causes great contraction of

* On the Ova of Man and Mammiferous Brutes, as they exist in the ovaries before impregnation; and on the discovery in them of a vesicle analogous to that described by Professor Purkinje in the immature egg of the bird. Read before the Royal Society, June 18th, 1835.

† Observations on the Ova of the Mammifera before and after impregnation, in reference to Dr. Martin Barry's "Researches in Embryology."

‡ On the first Changes in the Ova of the Mammifera in consequence of impregnation, and on the mode of origin of the Chorion. In Philosophical Transactions, Part ii. for 1837.

it, as well as of the yelk. The zona thus changed is what Meyer has mistaken for a different and distinct vitellary membrane.

§ 19. Bischoff comes to the conclusion already arrived at by the author of this report, that the yelk of the mammiferous ovum in the ovary is composed of a quantity of granules held together by a connecting substance, and *possesses besides the zona pellucida no other proper investment.* The zona pellucida, or rather the membrane on which the appearance of zona depends, therefore, if it must have a name, ought to be called *vitellary membrane.**

§ 20. *Germinal vesicle and germinal spot.* By the discovery of the germinal vesicle in the mammiferous ovarian ovum, the complete analogy between the latter and the ovarian ovum of the bird, &c. was established, and Baer's error regarding it dissipated. The correct view of the matter had been suspected by Purkinje, but he and Valentin had in vain searched for a germinal vesicle, and it was only on renewing their investigations after the announcement that such a vesicle had been discovered in the rabbit's ovum by M. Coste, that they, Wagner and others in Germany, were successful in finding it. M. Coste, therefore, as Bischoff observes, must, notwithstanding his very imperfect description and delineation of the germinal vesicle,be considered as its first discoverer. It is nevertheless, Bischoff continues, quite certain that the author of this report made the discovery independently, and demonstrated the vesicle in a much more definite and certain manner, inasmuch as by opening the ovum he exhibited it in an isolated state. (Kaninchen-Eies, p. 12, and Soemm. vol. vii, pp. 8 and 14.)

§ 21. Though he has made use of compression for demonstrating the germinal vesicle, Bischoff says that he succeeds with greater certainty by opening the ovum under the simple microscope by means of a very finely-pointed needle. This was the plan originally adopted by the author of this report. If the vesicle thus isolated, says Bischoff, be brought under the compound microscope, we see that it represents a simple cell with a very fine transparent and structureless membraneous wall with perfectly clear contents.

§ 22. The size of the germinal vesicle, Bischoff found pretty constantly to be in the mature ova of the rabbit about 1-600 inch. The size varies somewhat both relatively and absolutely, but within very narrow limits. It is relatively larger in small ova. The discrepancy in the statements regarding its absolute size given by different authors appears to depend more on the mode of examination than on actual difference. When compression has been used it appears of greater diameter than when observed in the perfectly unaltered state,—without the ovum being compressed or laid open.

§ 23. At one side of the germinal vesicle there is a small round dark spot discovered and described contemporaneously by R. Wagner and the author of this report. Wagner first gave it the name of "germinal spot," and recognized it to be of general occurrence throughout the animal series. This was a discovery of great value, and considered as such by both its authors, though it is to be confessed that the true import of the germinal spot became evident only after Schwann's application to animal structures of Schleiden's theory of development of vegetable structures from cells. In accordance with this theory, Wagner now calls it *nucleus germinativus*;—but more on this head below.

§ 24. Wagner says that he has sometimes observed in the mammiferous ovum the germinal vesicle with more than one spot, as in the ovipara. Neither Valentin nor Bischoff have ever seen such a case. The germinal spot of the mammiferous ovum is a disc about 1-3000 inch in diameter, composed of a finely-granulated substance, strongly refractive of light and attached to one part of the inner surface of the wall of the germinal vesicle which it causes to be more prominent externally there than elsewhere.

§ 25. As regards the position of the germinal vesicle in the yelk, it has been

* In the remainder of this Report, the name "vitellary membrane," followed by the word "zona" within brackets, will be employed to express the thick external membrane of the ovarian ovum.

already remarked by previous observers that it is found in immature ova more in the centre, in mature ova more towards the periphery of the yelk. Bischoff says that it does not appear to be in the yelk surrounded by any particular mass or formation, (*discus proligerus* properly so called,) as is the case in the bird's egg. Perhaps the true state of the case is that in those ova in which the yelk-granules and cells hold together in the manner above described, the germinal vesicle is imbedded in one side of the yelk-ball, and that in a definite manner as in a proligerous disc. The germinal spot, it is to be observed, is always towards the periphery, and is frequently the only part of the vesicle visible, the rest being covered by yelk-grains, a condition which can depend alone on the germinal spot being more prominent than the rest of the vesicle.*

§ 26. In his third series of Researches in Embryology, p. 531, Dr. Martin Barry says, under the head of preparatory changes in the germinal vesicle and germinal spot, that "the germinal vesicle does not 'burst,' 'dissolve away,' or 'become flattened,' on or before the fecundation of the ovum, as hitherto supposed. It ceases to be pellucid; and this perhaps is one cause of the mistaken views regarding it. But another cause is possibly a less transparent state assumed by the surrounding substance; and a third, is doubtless the almost entire absence of observations on mammiferous ova at this period." According to Dr. Barry, the germinal vesicle fills with cells, and these in their turn with the foundations of other cells, so that the germinal vesicle is gradually rendered nearly opaque, and becomes very much enlarged and flattened. The position of the germinal vesicle does not change, but it becomes more determinately applied to the investing membrane than in the immature ovum. The pellucid part of the altered germinal spot, at which the foundations of new cells arise, is directed towards the surface of the ovum. More particularly the part in question is directed towards an attenuated region or an orifice in the thick transparent membrane,—vitellary membrane, (zona.)

§ 27. From these statements of Dr. Barry, Bischoff dissents in the most decided manner. "Neither before," says Bischoff, (p. 15,) "at a time when I was endeavouring with the greatest care to make out the relations of the germinal vesicle in mature ova, nor yet since my attention has been again turned to the subject by Barry, could I observe the slightest indication of what he describes. I expressly instituted for the purpose examinations of the ova of a rabbit much in heat. Another experienced observer who assisted me did not succeed better."

§ 28. *First formation of the Graafian follicle.* Bischoff agrees with Henle in viewing the Graafian follicle in its early state when it has for its wall a simple structureless membrane as similar in nature to those vesicles which Henle and Goodsir find to be the elementary constituents of glandular structure.

§ 29. This view of Henle does not coincide with the representation given by Valentin of the development of the ovaries and Graafian follicles. The latter he describes as being formed within blind tubes, which originally constitute the structure of the ovaries, as the seminiferous tubes the testicles, but which become obliterated by the increasing growth of the Graafian follicles. Bischoff, however, says he has never been able to discover any such structure in the ovary as that which Valentin describes, notwithstanding every possible attention in the examination of the embryos of man, the cow, sheep, sow, dog, rabbit, hare, and rat.

§ 30. Henle's view is likewise not in agreement with what Martin Barry says on the subject. According to the latter, the first-formed part of the Graafian follicle, which he calls *ovisac*, is developed around the already existing germinal vesicle.

§ 31. Though Valentin differs from Henle and Bischoff in the account of the conditions under which the Graafian follicle is first developed, he appears to agree with them that the ovum is formed within the Graafian follicle, and not, as Barry states, the Graafian follicle around the rudiments of the ovum.

* "What first strikes the eye," says Henle, Allgm. Anat. p. 969, "is in general not the bright contour of the germinal vesicle, but the dark germinal spot."

§ 32. *First formation of the ovum.* Purkinje, the original discoverer of the germinal vesicle, conjectured that this might be the part of the ovum first formed. The observations of Baer and of Wagner on certain of the invertebrata showed that as regards these animals such is the case; and as regards vertebrata, Dr. Martin Barrŷ believed his observations warranted him in stating that in the rabbit and pigeon at least the germinal vesicle is the part of the ovum first formed.

§ 33. The following is Bischoff's account of the first formation of the mammiferous ovum: The fundamental part of the Graafian follicle being formed in the manner above described, contains a clear fluid, in which are suspended nuclei and granules, the latter quite similar to the subsequent yelk-granules. The size of the Graafian follicle at this period varies from 1-1000th to 1-333d of a Paris inch. On the inner surface of the *tunica propria* of the follicle, there is deposited a layer of endogenous cells as an epithelium. After this there is found in the centre of the follicle a nucleated cell perfectly similar to the germinal vesicle, which indeed Bischoff holds it decidedly to be. Around this vesicle are then found deposited those granules similar to yelk-granules, and that in greater quantity the more advanced the development is. At the next stage, when he was able to satisfy himself of the precise state of matters, Bischoff found in the Graafian follicle the ovum with all its essential parts, viz. vitellary membrane (zona), yelk, germinal vesicle, and its spot. The smallest follicles in which such an ovum could be distinguished measured 1-100—1-20th of a Paris inch in diameter.

§ 34. In the ova at this period, the vitellary membrane (zona) is very faint, and its external boundary not well defined. The yelk also is still clear. These parts, therefore, are difficult of being seen through the wall of the Graafian follicle, especially as it is no longer so transparent as formerly, in consequence of its *membrana propria* being now surrounded externally by a quantity of fibre-cells, from which is developed the cellular and vascular coat which subsequently forms the wall of the Graafian follicle. Hence Bischoff has not been able to perceive the formation of the vitellary membrane (zona). Everything, however, appears to him in favour of Valentin and Henle's opinion, that the yelk-granules are deposited around the germinal vesicle, and then become surrounded by the vitellary membrane (zona). Bischoff here repeats that in the formation of the ovum he has never seen in addition to the vitellary membrane (zona) a trace of any other membrane to which the name *vitellary* might be given, such as Valentin and Barry allege exists.

§ 35. The earlier the stage of development, the larger is the ovum in proportion to the Graafian follicle. The ovum indeed is at first almost closely embraced by the Graafian follicle, as the ovarian ovum of the bird is by its capsule. The earlier also the stage of development, the larger is the germinal vesicle in proportion to the ovum.

§ 36. *Position of the ovum and its component parts in reference to the theory of cells.* Schwann doubted whether the ovum should be viewed as a primary cell, its nucleus being the germinal vesicle, its nucleolus the germinal spot, and its contents the yelk-granules, or the germinal vesicle and its spot as standing in the relation to each other of cell and nucleus. The latter is the view which appears most consistent with all the circumstances of the case; the germinal spot has the size and form, and in relation to the germinal vesicle also the position, of a cytoblast, though direct observation in the higher animals has not shown that the germinal spot is first formed, and that around it is developed the germinal vesicle as a cell around its nucleus; but in insects, according to Wagner, the spot appears first.

§ 37. Adopting the view that the germinal vesicle and spot are respectively primary cell and nucleus, the signification of the yelk and its membrane remains to be accounted for. Bischoff coincides with Valentin and Henle in the view that the yelk and its membrane are deposit formations around the germinal vesicle, which thus, without actually being a nucleus, stands in the relation of one to the deposit formation around it. The exact mode in which the vitellary membrane (zona) is formed is still obscure. Bischoff conjectures that it results from the fusion of a peripheral layer of cells. Barry suggests (3d series, p. 538,) the consideration whether the "zona pellucida" may not be *formed* by a succession of his alleged vitellary membranes. He says further, it would appear that the ovisac is a great cell, as well as possibly the thick transparent membrane (vitellary membrane, zona.) The substance surrounding the germinal vesicle (the yelk of authors generally and of Barry's 1st and 2d series) in certain states exhibits changes similar to those presented by a nucleus, so that the substance in question seems to be a great "cytoblast."

§ 38. Is the part which the germinal spot plays concluded with the formation of the germinal vesicle around it, or has it still an important part to play? To the first question Valentin, Henle, Kölliker and others, to the second question Barry and Vogt, give the affirmative. Barry considers the germinal spot as the central point of new cell generations, a view which he extends to cell nuclei in general. Vogt considers the germinal spot not a nucleus, but a cell itself of the highest importance for the further development. This question will come to be considered further on.

### II. ON FECUNDATION, AND ON THE DISCHARGE OF THE OVUM FROM THE OVARY—CORPORA LUTEA.

§ 39. In many of the lower animals it is well known that actual contact of the seminal fluid with the ova takes place; that this contact is a necessary condition for fecundation has been proved by the experiments of Spallanzani and many others. As regards the mammifera, though there is less direct evidence in support of the thesis that for fecundation actual contact of the seminal fluid with the ova is necessary, still that evidence appears conclusive. In the first place the observations of Leeuwenhoeck, Prevost and Dumas, Bischoff, and others positively show that, *post coitum*, all the surfaces over which the ovum or ova must pass in their course from the ovary to the uterus become spread over with spermatozoa—evidence of the presence of semen,—so that mutual contact of the male and female generative elements must necessarily take place. Secondly, the experiments of Haighton, Blundell, and Bischoff, to say nothing of the practice of sow-gelders,* constitute evidence of a negative kind. They show that when by destruction of the female genital passages anywhere from the upper part of the vagina to the fimbriated extremities of the fallopian tubes, the access of the seminal fluid to the ovum in the ovary, or of the ovum to the seminal fluid in the genital passages, is prevented, impregnation never occurs.

§ 40. But the question arises, does the fecundating contact take place first in the ovaries, or not until the ova have been expelled from them and received into the Fallopian tubes? If analogy with what is the case among those animals in the impregnation of which the fact and the necessity of actual contact of the seminal fluid with the ova have been most directly proved, be adopted as a guide in the determination of this question it must be said that fecundating contact does not take place until the ova have been expelled from their capsules in the ovaries and received into the Fallopian tubes.

§ 41. This view was maintained by Prevost and Dumas, not however on the grounds of analogy, but because they never discovered spermatozoa beyond the Fallopian tubes. The fact discovered by Bischoff and Barry, however, that the semen penetrates to the ovary overturns the force of this argument of Prevost and Dumas, but does it prove, as Bischoff and Barry think, that fecundating contact first takes place in the ovary; that contact of the seminal fluid with the ovary is a necessary condition for fecundation?

§ 42. The rarity of the occurrence of spermatozoa on the ovary, as observed by Bischoff and Barry, does not appear very favourable to this view, but Bischoff accounts for this rarity by supposing that there is one particular time only at which spermatozoa are to be found on the ovary, and at which the observer is not always fortunate enough to make his observation.

* In the operation of spaying, the operator appears more particular about removing the fimbriated extremity of the Fallopian tube than the whole of the ovary.

§ 43. The best proof that contact of the seminal fluid with the ovary is a necessary condition for fecundation, would be that Graafian follicles burst only after such contact, or when there was reason to believe that such contact had taken place. But, although Bischoff says, that when the semen comes into contact with the ovary, the Graafian follicles, previously much distended, become still more so, and at last give way, there are numerous facts to prove that Graafian follicles burst independently of any contact of the semen with the ovary. To say nothing of those cases to which there will be occasion to return, in which Graafian follicles have been found burst independently of the access of the male; the experiments of Haighton and Blundell on rabbits show that though by obliteration of some part of the female genital passages, the access of semen to the ovary was prevented and in consequence of that impregnation, still Graafian follicles were observed to have burst, and *corpora lutea* formed *post coitum.*

§ 44. Cases of ovarian conception, admitting the occurrence of such,* appear to argue most strongly in favour of the fecundating contact taking place in the ovaries; but from the cases on record no very certain inference in regard to the question can be drawn: at the most it can only be said that an ovum capable of being impregnated, not being expelled from the ovary in consequence of the access of the male, but being by some cause retained in that organ, there receives the fecundating contact of the seminal fluid which may have penetrated so far, as it has been found to do in the dog and rabbit. Such an abnormal occurrence, however, would constitute no proof that fecundating contact ordinarily takes place in the ovary.

§ 45. There are thus no other means left of determining the question but by inquiring whether there be any changes in the proligerous disc, or in the ovum itself while still in the ovary, sufficient to show whether it has or has not been fecundated. But before inquiring into these points, the following merits consideration:

§ 46. *Mode in which the seminal fluid is conveyed along the genital passages towards the ovaries.* It appears pretty certain that at the moment of ejaculation, the seminal fluid is received directly into the uterus. Bischoff found few or no spermatozoa in the vagina of bitches and rabbits after coitus, but the uterus quite full. Within the female organs the seminal fluid does not coagulate into a thick and viscid mass, and the spermatozoa are observed to be particularly active. It may be concluded therefore that the seminal fluid may reach the ovaries partly by diffusion through the mucus on the genital passages, partly by the movements of the spermatozoa. The rate at which spermatozoa can move is, according to Henle, one inch in seven minutes and a half, a rate quite sufficient to account for the arrival of some out of the great numbers poured into the uterus, within the time after coitus, at which they have been found on the ovaries. There is another means which appears adapted to promote the progress of the seminal fluid towards the ovaries, and that is quick and progressive, but not peristaltic contractions of the uterus and Fallopian tubes from the vagina onwards, which have been observed by Bischoff immediately *post coitum* in bitches and rabbits, especially the latter.

§ 47. The vibratile cilia of the epithelium of the uterus and Fallopian tubes, as their movement is from the ovaries outwards, if they do not hinder, can contribute nothing to the inward progress of the seminal fluid. Weak in the uterus, the ciliary motions are active in the Fallopian tubes, and are well adapted to carry the delicate ovum along them to the uterus. Bischoff in general found ciliary motion stopped in that part of the tubes already traversed by the ova, but still brisk in the parts yet to be traversed. He likewise found spermatozoa only *before* not *behind* the ova in the course of the latter along the Fallopian tube. Wagner found no ciliary motions in a pregnant animal, nor in one which had just littered.

§ 48. *The changes in the cells composing the membrana granulosa and proligerous disc* observed by Bischoff and Barry, *post coitum*, around ova from much distended Graafian follicles, and which will be more particularly noticed further

* It will by and by be seen that the existence of ovarian conceptions is closely connected with the question of the development of the chorion.

on, might be adduced as proof of fecundation taking place in the ovary, but this cannot be admitted as evidence of the fact any more than the presence of the corpora lutea which form in the ovaries, when it was impossible seminal fluid could have arrived at them.

§ 49. *Alleged changes in the ovum.* Dr. Barry believes that there are changes in the ovum, which favour the supposition that the ovary is the usual locality in which the ovum is fecundated. "*Post coitum,*" he says (in his second series, p. 350, &c.) "before the discharge of the ovum from the ovary, the germinal spot, previously on the inner surface, passes to the centre of the germinal vesicle; the germinal vesicle, previously at the surface returns to the centre of the yelk; and the (alleged) proper membrane of the yelk, previously extremely thin, suddenly becomes thickened. To these changes, Dr. Barry, in his third series, adds, that the orifice which he alleges presents itself in the thick transparent external membrane,—vitellary membrane (zona)—as a change preparatory to fecundation, probably closes before the ovum leaves the ovary, he having in no instance seen it after the discharge of the ovum from the ovary.

§ 50. Of all the above alleged changes, the return of the germinal vesicle to the centre of the yelk, is considered by Dr. Barry as sufficient in itself to show that an ovum has undergone fecundation. *Ante coitum,* it is as *near* as possible to the surface of the ovum, as if to receive the contact of the semen; *post coitum,* it is withdrawn as *far* as possible from that surface.

§ 51. That such a process as this takes place has not been affirmed by any other observer. A little reflection on the small size of the ovum and the relative size of the germinal vesicle and yelk, will incline the reader to think with the author of this report that the alleged *migrations* of the germinal vesicle are a mere conceit, that it is absurd to speak of the germinal vesicle being seen in its passage *half-way* between the surface and the centre of the yelk. A few granules, more or less, on this or that side of the vesicle is all the difference that could really exist.

§ 52. Bischoff has never seen such migrations of the germinal vesicle, and as to internal changes of the germinal vesicle and spot, Bischoff says that he considers the statements of Barry on this head very doubtful. He several times found the germinal vesicle in ova at the period in question quite unchanged, without any of the alleged remarkable metamorphoses of its spot which, had they existed, could not have escaped his observation. He therefore decidedly contradicts this statement of Barry. Bischoff thinks it probable, however, that the germinal spot is the point of fecundation; if so, and if proximity to the surface be a necessary condition, it has been above seen that such exists, for the germinal spot, even when the vesicle is covered by the yelk grains, projects free at the surface of the yelk. But more of this below. What Barry says about the thickening of the proper membrane of the yelk is quite unfounded, seeing that no such membrane exists, as has been above shown, and as Bischoff again repeats in reference to this period.

§ 53. As to the alleged orifice in the thick external membrane of the ovum, this leads to an inquiry into the *nature of the action of the semen on the ovum.*

That the spermatozoa are an essential part of the seminal fluid, may be considered as an indisputable fact, proved by their existence in the secretion of the testes of all animals capable of generating, and their absence in unfruitful animals, but do they immediately and materially act in the process of fecundation? That they do, appears to be supported by the experiments of Prevost and Dumas with the seminal fluid of the frog deprived of spermatozoa by filtration, in which no fecundation took place. But without adducing an experiment of Spallanzani, attended with contrary results, (for it might be objected to it that there is no certainty that all spermatozoa were really excluded,) it may be said, that as a sort of coagulation takes place in semen, the filter may retain other matter besides the spermatozoa, and give passage merely to serum, which it can easily be understood will be as inefficient for fecundation as serum of the blood is for nutrition, secretion, &c.

§ 54. When the spermatozoa were first discovered, it was fancifully supposed that in fecundation they got into the interior of the ova and became the embryos.

It is known that Prevost and Dumas revived this notion in a modified shape. Dr. Barry however is the first, indeed the only person who has alleged that he has actually observed spermatozoa in the interior of ova. This was first in the case of a rabbit of twenty-four hours, and a second time at a somewhat earlier stage. (Phil. Trans. Part I, 1843.) He had previously asserted (3d series, p. 533,) that on one occasion, in an ovum of five hours and a half, he saw in the alleged orifice of the thick transparent membrane—vitellary membrane (zona)—an object very much resembling a spermatozoon which had increased in size. Dr. Barry states that in his recent cases of spermatozoa actually within the ovum, the above-mentioned alleged orifice was no longer visible.

§ 55. In regard to this point, Bischoff says, (p. 31,) that in the cases in which he found spermatozoa in bitches and rabbits on the ovaries, or in which the ova had just escaped from the ovaries, (he once found, he says, in one bitch two ova in the tube, and one on the ovary escaped from the newly-burst follicle,) he never saw either a fissure or opening in the vitellary membrane (zona,) nor of course, a spermatozoon penetrating into it. He therefore does not hesitate to doubt very much this observation of Barry.*

§ 56. "I must here," Bischoff says, "call to recollection the condition of the ovum. It is surrounded by a pretty dense layer of cells of the membrana granulosa and "discus proligerus," which, especially in perfectly mature ova, closely invest its vitellary membrane (zona). For these reasons I hold it impossible to observe such an alleged opening in the vitellary membrane (zona), and such alleged changes in the germinal vesicle. But any one practically acquainted with the matter must hold it utterly impossible to recognize here, under this mass of cells, a spermatozoon in any such opening. But it may be said that the observation might have been made after the ovum was freed from the surrounding cells. The manipulation necessary for this, however, would be sufficient to remove the spermatozoon, although it had really been present. It is much to be wished," Bischoff continues, "that Barry's statements on this point may not be too hastily adopted."

§ 57. "We find the ova in the tubes," Bischoff goes on to say, (p. 32,) "always covered with numerous spermatozoa, but I have never been able to satisfy myself of the presence of one of these in the interior of the ova. I have indeed, on two occasions, in compressing an ovum taken from the tube, by the compressorium under the microscope, observed, when the yelk-grains had escaped from the burst vitellary membrane (zona), a spermatozoon very distinctly among the yelk-grains, and it seemed to come out from the ovum. But considering, as above said, that the ovum is covered round with spermatozoa, and that these spermatozoa are difficult of removal on account of being inclosed in layers of albumen, mistake is here both very possible and very probable. Moreover, it ought not to be forgotten that the possibility of the entrance of spermatozoa into the ovum is very doubtful, notwithstanding Barry's allegations. On this point we may refer to the many previous observations of others, in which in *no animal* has any opening in the vitellary membrane been seen. And how would it be with those ova which are fecundated only when they have already become surrounded by an albuminous layer, as in fishes and frogs?"

If a spermatozoon, Bischoff concludes, really penetrate, it must do so in some other manner than that alleged by Barry.

§ 58. The orifices in the thick transparent membrane of the ova delineated by Dr. Barry, appear to have been clefts accidentally produced by pressure or other violence. In regard to figs. 167 and 168 of his 3d series, representing what he supposed the spermatozoon in the orifice, it must be admitted with Bischoff, that they are little calculated to make the observation probable. Supposing spermatozoa really in the ova, it must of course be understood that they get in

* The observation detailed in his 3d series of Researches. Bischoff, when writing this, was not acquainted with Dr. Barry's more recent alleged observation of spermatozoa actually within the ovum.

only after the bursting of the Graafian follicle. But then, according to Dr. Barry, the orifice in the thick external membrane — vitellary membrane (zona) —is closed.

§ 59. Even although Dr. Barry's recent observations be correct, it would be premature to admit them as proof that fecundation is effected by the direct and immediate agency of the spermatozoa.*

§ 60. The opinion most in conformity with all known facts is that the liquor seminis is the material, which entering the ovum by imbibition directly fecundates it. The spermatozoa are to be looked upon as being to the liquor seminis what the blood-corpuscles are to the liquor sanguinis, essential to the maintenance of the proper constitution of their respective fluids. Such an opinion was recently published by Valentin.

§ 61. Bischoff thinks that it is while in its ovarian capsule that the ovum is fecundated, and that the liquor seminis is imbibed through the walls of the follicle in order to get to the ovum; the author of this report is not prepared to deny this, but taking into consideration all the circumstances above passed in review, he is of opinion that there is no proof that fecundation takes place until the ovum escapes from the Graafian follicle and comes into direct contact with the semen.

The only unequivocal evidence of fecundation having taken place appears to be the remarkable subdivision of the yelk which occurs, but as will be seen not until the ova are in the Fallopian tube, a subdivision of the yelk similar to that which is known to be the first evidence of fecundation in the ova of those animals which admit of being directly observed after the application of the seminal fluid, (e. g. frogs, &c.)

§ 62. How long *post coitum* is it before the Graafian follicles burst? On this head there has been considerable difference in the statements of different observers. But it is pretty certain that the time varies in different species of animals, if not in the same. As regards the rabbit, Barry's numerous observations show that the average time is between nine and ten hours. Bischoff agrees in this. In the dog the time is variable but later. As regards man, nothing certain is known. It was supposed by Prevost and Dumas, that in the rabbit all the ova of one impregnation are not discharged about the same time. Bischoff agrees with Barry in contradiction of this.

§ 63. *State of the ovum from the period post coitum until it leaves the ovary.* The reader will perceive that several of the changes in the ovum mentioned in the following quotations from Dr. Barry, have been already considered in the discussions on the process of fecundation; but it is necessary here again to bring them under notice in correlation with the other changes which take place, or are alleged to take place, in the ovum at the period now under consideration.

§ 64. "From my observations," says Dr. Barry (2d series, p. 314, § 140; Phil. Trans., Part ii. 1839,) it appears that there is no condition of the ovum uniform in all respects which can be pointed out as that in which it is expelled; though the same observations lead me to conclude that plate v., figs. 96 and 97, present a state which is frequent when the discharge takes place, viz. the germinal spot has a central pellucid point; it is situated in the centre of the germinal vesicle; the latter has a dark contour, and perhaps a double membrane; around it is an ellipsoidal mass; the yelk is a fluid containing granules; the proper membrane of the yelk (?) has thickened, highly refracts light, and is often reddish-brown in colour; there is a minute space filled with a transparent and colourless fluid between this membrane and the thick transparent external membrane—vitellary membrane (zona); and finally the tunica granulosa (?) and retinacula (?) ("discus proligerus") present the appearance of incipient liquefaction.

§ 65. The following is the substance of what Dr. Barry says and delineates in

* The process of fecundation in plants has been thought to favour this view; but without any good reason in analogy.

his 3d series (p. 534, Phil. Trans., 1840,) regarding the "changes in the ovum immediately after fecundation, and before the ovum leaves the ovary."

In plate xxiii., fig. 169, he delineates an ovum of five hours and a half, in which the germinal vesicle was apparently undergoing the change of place towards the centre of the ovum. The vesicle had begun to regain its globular form. The point of fecundation, however, was still visible at the periphery; whence, and from the unclosed state of the fissure in the thick transparent external membrane—vitellary membrane (zona),—he is disposed to think the ovum had not been long fecundated, for the point of fecundation is subsequently seen to occupy the centre of the germinal vesicle, and soon after fecundation the orifice in question is no longer seen.

§ 66. In his 3d series Dr. Barry, as was above mentioned, drops the expression "yelk" and employs that of "substance by which the germinal vesicle is surrounded." The same process of cell-formation which in the mature ovum he alleges had begun in this substance before fecundation, he describes as being continued after fecundation. In the ovum, fig. 170, the germinal vesicle having receded from the surface into the interior of the ovum, had become closely surrounded by a layer of cells, each of which presented a remarkably opaque nucleus. Subsequently this nucleus seems to resolve itself into cells, and the same origin of new cells appears to take place in its interior as that which he had described in a former page (noticed in this report under the head of Germinal Vesicle). It appears also that new layers of cells come into view internal to the layer just described, when a succession of the same changes takes place as those already mentioned. Layer after layer of cells makes its appearance in the interior,—often seen to have become circumscribed by a proper membrane,—while cells occupying a more external situation undergo liquefaction.

§ 67. The cells of the (alleged) tunica granulosa undergo, immediately after fecundation, a remarkable alteration in position, size, form, and internal condition. Being first loosened and made less adherent to one another, they become club-shaped, greatly elongated, and connected with the thick transparent membrane —vitellary membrane (zona)—by thin pointed extremities alone. They present in their interior, at the large extremity, a pellucid space apparently corresponding to the *enlarged nucleolus* of other cells. This space is surrounded by dark globules. Subsequently there is seen, instead of this pellucid space, a cell-like object which contains a colourless and transparent fluid; but does not exhibit any proper nucleus or "cytoblast," and the surrounding globules become scattered. At a later period, these cells of the tunica granulosa are found filled with other cells.*

§ 68. Bischoff tells us, that several years ago he observed that ova from much distended Graafian follicles, *post coitum*, presented a peculiar condition of the cells of the *membrana granulosa*, and especially of those of the "*discus proligerus*," around the ovum; an appearance which he never saw in other ova, although apparently quite mature. The cells appear larger, more transparent, the nucleus is then more distinct, and they adhere more closely together, so that when the follicle is opened they do not become scattered in the fluid, but the membrane *in toto* escapes as a viscid jelly-like mass. The cells of the disc become at first club-shaped, their pointed extremities being attached to the zona or thick external membrane. The clear nucleus is very distinct in them, but Bischoff never saw it as Barry represents it, nor yet filled with young cells. The cells afterwards extend into a spindle-shape, whereby the ovum acquires a peculiar stellate appearance. This appearance Bischoff found constant in dogs and rabbits, and as Barry has observed it also, it may be considered as certain.

§ 69. In the appearance of the ovum in other respects, Bischoff found nothing changed except that the vitellary membrane (zona) was generally somewhat thicker, and the yelk appeared quite full and dark. In Soemmerring, he repeats,

* The second and third of these conditions, however, so far as Dr. Barry's observations have extended, are not generally met with until the ovum has been discharged from the ovary.

that he never could observe any vitellary membrane distinct from the zona, and in this respect contradicts Barry in the most emphatic manner. He never saw, as above mentioned, any orifice or fissure in the vitellary membrane (zona), although he purposely examined six ova of a bitch eighteen hours and a half post coitum, after he had freed them from the cells of the disc. As little did he ever see the cell-layers of the yelk mentioned by Barry, but the yelk always appeared finely granular. On every occasion he looked most carefully for the germinal vesicle. Sometimes he found it, sometimes he did not. He observed, that in consequence of the greater density and coherence of the yelk, it was more difficult to observe and extract the vesicle than in other cases; so that it might have existed in those ova in which he did not find it. In those cases, however, in which he did find it, both it and its spot appeared quite unaltered, without any of the remarkable metamorphoses of the spot mentioned by Barry; which, had they existed, could not well have escaped his notice, especially in the case of the bitch above mentioned. The objection, that in the case in question the ova might not have been destined to escape from the ovary on this occasion, Bischoff meets by saying that the ova were at least perfectly mature; and Barry, as has already been seen, affirms that the changes of the germinal spot and germinal vesicle present themselves *ante coitum* and independently of it.

§ 70. Bischoff does not hesitate to express it as his present conviction, although circumstances render the proof very difficult, that the germinal vesicle at the end of the period between coitus and the escape of the ovum from the ovary in general dissolves. The moment of disappearance, however, is not definite; and he thinks it probable that the vesicle may not disappear until the ovum is in the Fallopian tube. From observations on other animals it is known that the germinal vesicle disappears sometimes before, sometimes after, the escape of the ovum from the ovary; sometimes before and sometimes after coitus; what alone is constant is that in ova in which development has actually commenced, the germinal vesicle is no longer found. The same thing he thinks holds in regard to the mammifera.

§ 71. *Corpora lutea.* It has been above seen that the experiments of Haighton and Blundell on rabbits show, that though by obliteration of some part of the female genital passages, the access of semen to the ovary was prevented, still Graafian follicles were observed to have burst and *corpora lutea* formed after the accomplishment of coitus. In similar experiments Bischoff found pretty-fully developed *corpora lutea,* which he says could not well have depended on a previous fecundation. In the page preceding that in which Bischoff mentions this experiment (p. 43,) he says that he has never found fully-formed corpora lutea without coitus having taken place; from which it might be inferred that he must suppose that the excitement of coitus alone determines the formation of corpora lutea, and that independently of the contact of semen with the ovary; but in Soemmerring (p. 35) he is found saying, in reference to man, that a corpus luteum is no proof of previous coitus, because the bursting of the Graafian follicle, of which he here says the corpus luteum is a proof, is evidently often the result of other causes, such as sexual excitement in general, and menstruation.

§ 72. There are indeed numerous facts to prove that Graafian follicles burst independently of the access of the male. The researches especially of Dr. Robert Lee, and subsequently those of MM. Gendrin and Negrier, have rendered it extremely probable, if not certain, that in the human female the phenomena of menstruation are intimately connected with the periodical spontaneous bursting of Graafian follicles.

§ 73. But the question here arises, is the corpus luteum, which is formed by the bursting of a follicle independently of the access of the male, similar to a corpus luteum which is formed in consequence of impregnation? Bischoff says in regard to the rabbit, as has been above seen, that he never found fully-formed corpora lutea without previous coitus; but he adds, that he has several times seen Graafian follicles filled with blood, but containing no ovum, independently of coitus, and he thinks that the Graafian follicle does not burst, but only swells and becomes filled with blood, whilst the ovum is absorbed, and a false corpus luteum formed. It is to be remarked that Bischoff, though he here speaks of fully-formed corpora lutea and false ones, does not give any mark of distinction between them; and from what he says in Soemmerring (above quoted) it might be inferred that a corpus luteum is the same, whether resulting from, or independently of, the access of the male.

§ 74. The observations of Dr. Lee show that there is a difference between the corpora lutea formed under the two different conditions above referred to. In the false corpus luteum the yellow substance is at the inner surface of the wall of the Graafian follicle, and does not surround it, as is the case in true corpora lutea. In a specimen of false corpus luteum, which the author of this report examined with Dr. Lee, there was yellow matter deposited in the coats of the Graafian follicle, which were spongy and thickened. The cavity of the follicle was distended with coagulated blood: the inner layer had a peculiar wrinkled appearance, similar to what Baer has represented in his figure of the true corpus luteum, and the suspicion could not be avoided that it was a corpus luteum of this kind which Baer represents in his work 'De Ovi Mammalium et Hominis genesi,' as a true corpus luteum.

§ 75. Baer's opinion that the corpus luteum consists in a growth inwards of the inner layer of the Graafian follicle, is supported by Bischoff in opposition to opinions entertained in this country, viz.—that the yellow substance is contained between the two layers of the wall of the Graafian follicle according to Dr. Montgomery; or that it is outside both according to Dr. Lee. Barry's statements appear to favour Montgomery's view, though in reality he agrees essentially with Baer, and that apparently without knowing it. Barry considers his "*ovisac*" as the inner layer of the Graafian follicle; now Baer, in speaking of the inner layer of the Graafian follicle undergoing the change to form a corpus luteum, could not have referred to Dr. Barry's "*ovisac*," inasmuch as he did not know of the existence of such a structure, but to the inner layer of the cellulo-vascular wall of the Graafian follicle —the *covering of the ovisac* of Barry. All this indeed Barry is aware of; but in § 157 of his second series, unnecessarily regrets to find himself expressing an opinion at variance with that of Baer; and in his subjoined note coincides with Montgomery in his view of the corpus luteum, but inaccurately, according to his own principles, inasmuch as Montgomery speaks of the layers of the Graafian follicle in the same sense as Baer.

§ 76. The fact is, Dr. Barry's "*ovisac*," or, as Bischoff calls it, *tunica propria** of the Graafian follicle exists only at the commencement of development, and is not to be found afterwards. "From my observations on the formation of the Graafian follicles," says Bischoff, p. 45, "I have indeed admitted a *tunica propria*, which becomes overlaid externally by a layer of fibres, and with this represents the follicle. But I have never found that this *tunica propria* admits of being separated as a distinct layer of the follicle, but have found reason for admitting it only in the process of development." Barry says (2d series, p. 317, § 154), that in a few hours after the ovum has been discharged from the Graafian follicle, the "ovisac" may be readily removed by pressure from the burst Graafian follicle; but in § 155 he says, that at the end of several days the primitive ovisac is no longer met with in the ovary; and in a note he remarks, that he does not know whether in the interim the ovisac has been absorbed *in situ* or first expelled, but in the hog he has found what seemed the remains of ovisacs in the infundibulum. In reference to these statements Bischoff observes, that he has never seen that any *tunica propria* becomes loose from the rest of the follicle after the expulsion of the

* The terms *ovisac* and *tunica propria* are employed in the descriptive and illustrated catalogue of the museum of the Royal College of Surgeons, vol. v. in a different sense from that in the text. *Ovisac* is used for the whole Graafian vesicle or follicle; *tunica propria* is applied to the cellular and vascular wall of the Graafian vesicle or Barry's "covering of the ovisac;" whilst *ovarian vesicle* is used for what is referred to in the text under the names of *ovisac* of Barry or *tunica propria* of Bischoff.

ovum. The gelatinous-like mass found in the follicle in the first period after the escape of the ovum, he asserts, is by no means the tunica propria of the follicle or the ovisac, as it has been seen Barry thinks, but the fluid of the follicle and membrana granulosa which had not all escaped, changed and become more consistent, and which by great development of its cells has been converted into a viscid coherent mass." Barry's own figure 98, plate 5, is corroborative of this assertion of Bischoff.

§ 77. To return to Montgomery and Lee. The cases adduced by these gentlemen unequivocally show, that human true *corpora lutea*, at least, are not growths of the inner layer of the Graafian follicle—the inner layer in the sense of Baer and not of Barry—for in such corpora lutea not long after conception the inner layer is found but little changed, and not at all the seat of the yellow growth.

§ 78. The yellow substance, it is to be observed, is a new formation, and not a *conversion* of the cellular tissue of any part of the wall of the Graafian follicle, as appears to be the opinion of Baer, Barry, Bischoff, and the author of the notices in the Catalogue of the Museum of the College of Surgeons. The designation of the corpus luteum by the latter, as the "thickened parenchymatoid proper tissue, or tunic of the ovisac," must be held to be erroneous. From the statements in the Catalogue regarding the corpora lutea in the College of Surgeons' Museum, it cannot but be admitted, that the preparations support the views of Montgomery and Lee, so far as these two gentlemen concur, rather than those actually given in the Catalogue.

§ 79. As above stated, Montgomery is of opinion that the yellow substance is deposited between the two layers, into which the cellulo-vascular wall of the Graafian follicle may be separated by dissection, whilst Lee maintains that the yellow substance is wholly outside both, and that any appearance of membrane between the yellow substance and the stroma of the ovary, which may in some cases present itself, is due simply to condensation of the neighbouring part of that stroma. From the examination he made of the very recent corpus luteum described by Dr. Lee in the Medico-Chirurgical Transactions a few years ago,* the author of this report was led to concur entirely in this view of Dr. Lee; other cases which he has examined support the same view; and it may be observed that the statements in the description of the corpora lutea in the Catalogue of the College of Surgeons are unequivocally to the same effect.

§ 80. It may not be uninstructive in conclusion, to inquire how it is that Baer, Bischoff, and others, should have viewed the corpus luteum as a growth of the inner layer of the Graafian follicle. In regard to the human corpora lutea described by Baer: in several cases the author of this report has verified the correctness of Baer's description; but in all there was no doubt, from the history, that the corpora lutea had been developed independently of impregnation. As to Bischoff's account of the corpora lutea in the rabbit, it will be found on reexamination that the *whole wall* of the Graafian follicle has been contracted together and pressed by the increasing growth of the substance of the "corpus luteum" situated really between it and the stroma of the ovary towards the most prominent part, where by being inverted through the aperture in itself by which the ovum escaped, it forms the pouting papilla-looking body described by De Graaf, Cruikshank, and Barry. The description, in fact, given by the latter, viz.: "The mammillary process appears to consist solely of an inverted portion of the vascular and spongy substance which previously constituted the covering of the ovisac," completely bears out what is here contended for,† and does not in the slightest degree warrant the conclusion he himself comes to, and which he designates as *obvious*, viz.

* Valentin (Repertorium, vol. vi.) mentions his having observed a similar case to this. In a woman who died in the fourth month of pregnancy, there was what he calls "a hollow cyst, such as is described by Lee and Jones" in the corpus luteum.

† The same process appears to take place in the formation of the corpora lutea in the cow, &c. and is the explanation why a central cavity is not found as in human corpora lutea, but simply the smooth circular depression on the most projecting part.

that *the covering of the ovisac becomes the corpus luteum.* (2d series, p. 318, § 156).

### III. Changes which the ova undergo in their passage along the Fallopian tubes.

§ 81. Von Baer was the first who, with a knowledge of the ovarian ovum of the mammifera, found ova in the Fallopian tubes. This was in a bitch. The conclusion he came to from his observation was, that the ova undergo but little alteration in their passage through the tubes. The author of this report, the next observer in order of time to Baer, described ova from the Fallopian tubes of the rabbit, and showed, on the contrary, that in that animal at least they undergo very remarkable changes. The subsequent and more extended researches of Barry and Bischoff, while they confirm generally the author's statements, supply more detailed information.

§ 82. It is purposed first, to consider under one head *the changes in the envelopes,* then under another, *the changes in the vitellus, including the germinal vesicle,* and after that to examine them in their correlations.

§ 83. *On the changes in the envelopes.* Baer represents the thick external membrane of the ovarian ovum as still constituting the external envelope of the ova, which he found in the Fallopian tubes of the bitch. And Bischoff (writing in 1838) says: "it is quite certain that the ovum receives no new external investment in the tube; it acquires no albuminous layer and cortical membrane like the ovum of ovipara; and I can therefore state positively, that the outer covering of the ovarian ovum also remains the outer covering, (always excepting the decidua,) a circumstance which had been spoken of as probable by Baer, Wagner, Coste, and others. Wharton Jones," continues Bischoff, "the only person who pretends to have observed this formation of albumen and cortical membrane around the ovum, was probably deceived by the 'discus.' He has either not been sufficiently acquainted with it from the ovary onwards, or no longer expected it in the fecundated ovum, (as indeed it does disappear at a later period,) and has taken its granules for the albuminous layer forming around it." (Wagner's Physiology, Leipzig, 1838, p. 96, § 69.—Willis's Translation, London, 1841, p. 141.)

§ 84. Dr. Barry, (writing in 1838 also,) not less positively, though in an indirect manner, contradicts the author of this report, inasmuch as he speaks of the thick transparent external membrane of the ovarian ovum, as continuing in the tubes and uterus the external membrane. Having, as already seen, admitted without any foundation a vitellary membrane, in addition to the thick transparent external membrane of the ovarian ovum, Dr. Barry discusses this thick transparent external membrane in a section of his 1st series, entitled "the true chorion, a structure superadded within the ovary in the class mammalia."*

§ 85. The observation by the author of this report, thus so decidedly contradicted, directly by Bischoff, indirectly by Barry, was that in a rabbit three days *post coitum,* he found in the Fallopian tubes, near where they enter the horn of the uterus, ova differing very remarkably from ova as they exist in the ovaries before impregnation; inasmuch as they each presented, in addition to the component parts of the ovarian ovum, a thick gelatinous matter surrounding it, similar to what is observed around the ovum of the frog in the oviduct.†

§ 86. When Bischoff wrote the remarks above quoted from Wagner's Physiology, his observations had been confined to dogs. Observations since made on

* It was from the views he propounded in this section, that Dr. Barry was led to substitute the word *ovum* for *ovulum,* which was that originally employed by Baer to designate the body he discovered in the ovary. But as the views were erroneous, so was the reason for the change of name founded on them. It is to be remarked, however, that the change of name from *ovulum* to *ovum,* which the author of this report was the first to make, is correct, and that for the reasons adduced by him.

† On the first Changes in the Ova of the Mammifera in consequence of impregnation, and on the mode of origin of the Chorion. In Phil. Trans. Part ii. for 1837.

rabbits have shown him "that the ovum of the rabbit, like that of many ovipara, becomes surrounded in its progress along the Fallopian tube with a layer of albumen." Barry's* subsequent observations have also convinced him that in the ovum in the Fallopian tube, "an outer membrane becomes visible, first as a dark circle around the thick transparent membrane," then by the imbibition of a fluid having "no small consistence," it becomes distended, and thus separated from the thick transparent membrane, so that the latter now appears as if surrounded by a gelatinous-looking substance.

§ 87. The existence of an additional investment† around the ovum of the rabbit in the Fallopian tube being thus admitted, and that by persons who previously denied it, it remains to inquire into its nature and mode of origin. It may in the meantime be observed that the author of this report indicated this additional investment as the basis of the future chorion. In this view he was followed by Barry,‡ but as will be seen to a certain extent only by Bischoff.

§ 88. As above seen, the author of this report described the additional investment around the rabbit's ovum from the tube, as a gelatinous-looking matter, similar to that presented by the laid ovum of the frog. Dr. Barry objects to this view, saying that the additional investment consists of a membrane having a sensible degree of thickness, retaining a fluid which is interposed between it and the thick transparent membrane. In proof of this he adduces cases in which he observed that when the thick transparent membrane was ruptured by crushing, and not the additional investment, the contents of the ruptured membrane (yelk) proceeding no further than the dotted line, (See his fig. 128, pl. viii,) showed this to be the inner surface of a thick membrane, which had been concealed by an equal refracting power in the fluid interposed between it and the thick transparent membrane. In accordance with his view of the additional investment now stated, Dr. Barry in all his figures employs a dotted line to indicate *diagrammatically* the distinction between the fluid and its retaining membrane, which together form, as he alleges, the additional gelatinous-looking investment of the ovum in the tube.

§ 89. In reference to these views of Barry, Bischoff observes: "I hold it an easy matter to determine, that we have here to do with a gelatinous-like substance, and not with a fine membrane inclosing a fluid. And for these reasons:

*a*. "We cannot by microscopical examination convince ourselves of the membranous nature of the outermost limits of the said structure.

*b*. "When pressure is applied, there is never produced the effect which would be exhibited on a fine bladder filled with fluid, but that which was to be expected from the crushing of a gelatinous-like matter.

*c*. "In the most certain manner however, we convince ourselves by dissecting the ovum with a fine needle, under a good simple microscope. By this means (the same originally employed by the author of this report,) we can cut off segments of every description from this layer, which according to Barry's view had been impossible. I have adopted this procedure not only as a proof of the nature of the layer, but also frequently for the purpose of cleaning the zona, in order to be able to examine more closely the contents of the ovum. There is no small difficulty in removing this gelatinous-like mass from the ovum with the needle. In short, I can say that there is scarcely any point of my subject on which I am

* It is to be observed that Bischoff still maintains that in the dog no albumen forms around the ovum in the tube. If this be found by further observation to be really the case, Bischoff's error consisted in applying too hastily to the rabbit what he had found in the dog; Barry's error, on the contrary, is due to inaccurate observation, inasmuch as rabbits were the subjects of his researches.

† It is to be remarked that Cruikshank must have observed this additional investment. It, in fact, appears to be what he designated as chorion; but the author of this report was the first who, with a knowledge of the ovarian ovum, discovered it, and recognized it as a superaddition analogous to the gelatinous-looking matter which the ovarian ova of the frog acquire in their passage through the oviduct.

‡ In the quotations from Barry it will be observed that the word *chorion* is used to express the additional investment.

so certain as on this. The albuminous investment is elastic and yielding to pressure; that it consequently may sometimes give rise to an appearance such as is represented by Barry in figs. 128 and 130 of his 2d series, in which the yelk mass is effused between the zona and the albuminous layer, is not to be wondered at."

§ 90. As to the origin of this gelatinous looking investment of the ovum: two observations were adduced by the author of this report, to show that it is first acquired by the ovum in the ovary, from a change of the granules of the "proligerous disc" immediately surrounding it, but that in the Fallopian tubes it swells out, and acquires a greater diameter, as the corresponding part of the frog's ovum does when extruded, by the absorption of the water and seminal fluid with which it comes into contact.

§ 91. From the concurrent testimony of Bischoff and Barry, however, it would appear that the addition is first made to the ovum in the Fallopian tube, and that although the cells of the "proligerous disc" around the ovum in the ovary continue to invest it after its arrival in the tube, they are, Bischoff says, eventually entirely separated from it, and contribute nothing to the formation of the gelatinous-looking investment. Bischoff never saw any such layer of gelatinous-looking matter around the ovum in the ovary, as the author of this report described in the two observations above referred to, and as Bischoff never found ova still in the ovary forty-one and forty-eight hours post coitum, as is alleged to have been the case in those observations, Bischoff considers that the author of this report has erred, misled probably by the contents of the Graafian follicle, which at this time have become dense and gelatinous-looking.

§ 92. Bischoff and Barry differ however, as to the mode of origin of this investment.

Dr. Barry in his 2d series described it as *rising* from the thick transparent membrane of the ovum—vitellary membrane (zona)—in the Fallopian tube, and imbibing fluid, and thus becoming distended. In fig. 53, pl. ix. 2d series, Dr. Barry delineates an ovum of seventeen hours from the middle of the Fallopian tube, showing the mode of origin of the chorion. This membrane is rising from the thick transparent membrane or "zona pellucida," surrounded by part of the tunica granulosa. The interpretation which might be given of this figure is, that the cells of the "discus" were coalescing to form the gelatinous-looking investment. This Dr. Barry himself thinks probable at p. 342 of his 2d series, but in his 3d series, though he finds that it is formed of cells, it is not of the cells of the "disc" brought from the ovary. They are much more minute, and have a very different interior and form. Successive additions of such cells collect around the thick transparent membrane and coalesce. Imbibition of fluid and consequent distention then take place. Having thus in his 3d series on Embryology, determined that the chorion is formed of cells arising in the oviduct, Dr. Barry is found in his 1st series on the Corpuscles of the Blood, announcing that the chorion is formed of cells which are altered corpuscles of the blood! an announcement for which he truly apprehended physiologists were not prepared.

Bischoff describes the new investment on its first appearance, as a very thin perfectly transparent layer around the ovum, of a gelatinous-looking substance, which might easily be overlooked, but does not say how this substance is first formed. The investment increases in thickness as the ova proceed in the tubes. It has a stratified texture, and deserves in every respect the designation of white of egg. From this it appears that Bischoff supposes that the investment of the ovum under consideration acquires its thickness by the addition of succeeding layers like the albumen of the bird's egg.

§ 93. If the chorion be recognized as an essential part in the development of the ovum, and if it be admitted that, in the rabbit at least, the additional gelatinous-looking investment has an important share in the composition of the chorion, it will be seen that an ovarian conception, and the opinion according to which the formation of the additional gelatinous-like investment does not take place until the ovum has arrived in the Fallopian tube are incompatible; either an ovarian conception cannot take place in the rabbit, or the additional gelatinous-like investment may be formed in the ovary. If Bischoff's observations, however, be admitted as correct in regard to the ovum of the dog, and his conjectures in regard to the human ovum, viz., that there is in them no such additional gelatinous investment, but that the basis of the chorion is the vitellary membrane (zona), the same difficulty does not occur.

Though the author of this report is not disposed to put much confidence in the two observations above objected to by Bischoff, in consequence of the unfavorable circumstances under which they were made, still it is evident, from the difference in the statements of Barry and Bischoff on the point, that further observations than those yet adduced by them are required to determine the place and mode of origin of the new investment. Barry found it on an ovum of forty-one hours, and advanced only one inch in the Fallopian tube. Here the difference between Barry and the author of this report is merely as to the place where the ovum was found. It remains to be proved that ova are never found in the ovary so late as forty-one hours.

§ 94. In their progress along the Fallopian tubes, the ova are found to become double, or even more than double the size of ovarian ova. This increase in size is principally due to the addition of the gelatinous-looking investment. The vitellary membrane (zona) only becomes a little thicker. An accumulation of fluid is observed between it and the yelk, but the room for this fluid is supplied rather by the condensation of the yelk than the distention of its membrane.

§ 95. In ova in the Fallopian tubes, Bischoff says he never could perceive, any more than he had done in ova in the ovaries, any proper vitellary membrane in addition to the zona. Barry, still adhering to his notion that such a membrane exists, says that after having suddenly thickened, and being remarkably distinct, it disappears by liquefaction in the tube; so that the yelk is now immediately surrounded by the thick transparent membrane (zona pellucida) of the ovarian ovum.

§ 96. *The changes in the vitellus, including the germinal vesicle*, now come to be considered. A process of division of the yelk into more and more minute and more and more numerous, globular masses in a geometrical progression with the factor two, has been found to accompany the first development of the ova in many animals. In regard to the first discovery of this process in the ova of the mammifera, Bischoff quotes a passage,* and refers to a figure, (Fig. iii,) in Baer's 'Epistola,' which show that Baer had observed in the ova from the Fallopian tube of the bitch, the yelk undergoing a *resolution into globular segments ;* though the nature of the appearance escaped him, viz., its analogy to that division and subdivision of the yelk of the ovum of the frog after impregnation, first discovered by Prevost and Dumas.

§ 97. The nature of the appearance, however, did not, as Bischoff asserts, (Soemmerring, p. 58,) entirely escape the author of this report, the next observer in the order of time ; he observes in Part ii of his paper (Phil. Trans. 1837,) entitled "on the changes in the vitellus," that what he has to say regarding the changes in the vitellus in consequence of impregnation, relates chiefly to the ova of the batrachian reptiles, and is added merely for the purpose of throwing some light on the changes which take place in the yelk of the ova of the mammifera, previously to the commencement of the evolution of the embryo. Having determined, as he thinks, that the disappearance of the germinal vesicle is prior to, and not dependent on impregnation, he asks what is the first change which takes place in ova, in consequence of impregnation ? In answer to this he says that of all ova, the ova of the frog are those in which such change can be most directly observed, and that in them the breaking up of the surface of the yelk as described by Prevost and Dumas is the first change he has seen. He then goes on to state, as the result of this breaking up of the surface of the ovum of the frog, that the blasto-

* Medium tenet globulus sub microscopio penitus opacus, *superficie non lævi et æquali, sed granulosa, totus enim globulus e granulis constat dense stipatis,* membrana cingente vis conspicua. Globulum circumdat, interjacente spatio pellucido arcto, peripheria quædam stratu tenui granulorum minimorum obtecta. Post nycthemeræ macerationem hujus pulveris majorem partem sejunctam inveni ; quo facto membrana continua et simplex venit in lucem......... Mira est ovorum nostrorum parvitas. Quæ sub microscopio metitus sum 1-15 lineæ partem tantum diametro explebant. (p. 11.)

derma, consisting of an aggregation of clear globules, different from those of the rest of the yelk is now fully formed. After this, he says the change which takes place in the yelk of the bird's egg appears to be limited to the neighbourhood of the cicatricula. In the ovum of the mammifera, there being little more than a blastoderma to be formed, the whole of the vitelline grains undergo a change, and are resolved into a spherical blastoderma, presenting the same peculiar friable and globular texture as the blastoderma of the egg of the newt, frog, bird, &c.

What was here merely indicated by the author of this report, has been fully demonstrated and traced out by Bischoff and, though with erroneous interpretations, by Barry also, in the mammifera. It is in the invertebrata, however, especially viviparous entozoa, that the process has been most successfully traced, as will be seen below.

§ 98. Before considering this remarkable process, which, according to Bischoff, commences about the time the ovum enters the second half of the tube, according to Barry, between his "fifth and sixth stage" of development, the ovum being then about the middle of the tube, it will be proper to premise an account of previous changes spoken of by Barry and Bischoff in the first half of the tube.

§ 99. In his second series Barry states, that in an ovum in the "third stage" of development, found at the distance of one inch from the infundibulum, the germinal vesicle was visible in the ellipsoidal mass that occupied the centre of the fluid yelk. This latter was obscurely granulous. In an ovum of the "fourth stage," also about an inch from the infundibulum in the Fallopian tube, the yelk was also obscurely granulous, but the germinal vesicle was no longer seen. In an ovum at the "sixth stage," in which the alleged vitellary membrane has disappeared, there no longer existed a granulous yelk.* In the ovum delineated (fig. 106), the thick transparent membrane was filled with a transparent and colourless fluid, which, Dr. Barry says, it may be proper to designate the yelk.

§ 100. In his "third series," it has been already seen, Barry states among the "changes in the ovarian ovum preparatory to fecundation," that the germinal vesicle becomes filled with cells, and these again become filled with the foundations of other cells, so that the vesicle is thus rendered almost opaque, enlarged and flattened. This development of cells within the germinal vesicle proceeds from the germinal spot. In the centre of this spot there is invariably present at a certain period a dark point which enlarges, and is then found to contain a cavity filled with pellucid fluid. This central portion of the altered germinal spot, with its pellucid cavity, remains at that part of the germinal vesicle which is directed towards the surface of the ovum, and towards the surface of the ovary. At the corresponding part, the thick transparent membrane of the ovum, it has been already seen, Dr. Barry alleges, appears in some instances to have become attenuated, in others also cleft. Subsequently, the central portion of the altered spot passes to the centre of the germinal vesicle; the germinal vesicle regaining its spherical form, returns to the centre of the ovum, and a fissure in the thick transparent membrane is no longer seen. From these successive changes, it has been seen Dr. Barry infers that fecundation has taken place, and this by the introduction of some substance into the germinal vesicle from the exterior of the ovary. It may, he says, also be inferred that the central portion of the altered germinal spot is the point of fecundation. In farther proof that such is really the case, Dr. Barry affirms that there arise at this part two cells, which constitute the foundation of the new being,—that is the germ. The nucleus in each of these twin cells which together constitute the germ, undergoes essentially the same changes as those presented by the germinal spot, but seems to pass sooner than the centre of the altered germinal spot to the interior of its cell. These two cells which constitute the germ enlarge, and imbibe the fluid of those around them, which are at first pushed farther out by the two central cells, and subsequently disappear by liquefaction. The contents of the germinal vesicle thus enter into the formation of two cells. The membrane of the germinal vesicle then disappears by lique-

* Barry now questions the analogy which this term implies. He calls it, as above seen, "the substance surrounding the germinal vesicle."

faction. While the changes just described are in progress within the germinal vesicle, membranes form and then disappear successively around the layer of discs or cells by which this vesicle is surrounded (i. e. the yelk). Very soon, however, the substance they invest (i. e. the yelk), has wholly disappeared, and its place is occupied by colourless transparent fluid. (p. 538, § 380.) In this fluid are often found solitary cells, the remains of the substance just referred to. It is also very common to meet with these solitary cells in previous conditions of the ovum before the last of the membranes (alleged vitellary) has disappeared.

§ 101. In the first part of the Fallopian tube, according to Bischoff, the yelk has still quite the same appearance as in the ovary, uniform, finely granulous, and entirely filling the zona. A little further on, however, it no longer fills the zona, but is observed to be contracted, leaving between it and the zona a space in which there is a transparent fluid, in which for the most part two granules, often different in size, are seen swimming. Bischoff sought in vain for the germinal vesicle; neither under the compressorium, nor by opening and dissecting the ovum with a fine needle, could he observe anything like it. Sometimes only he thought he saw in the interior of the yelk a speck clearer but smaller than the germinal vesicle, but he never could come to any decisive conclusion regarding it. In these dissections of the ovum, however, he remarked that the yelk had evidently increased in coherence. By the action of water on the ova, the yelk which previously was contracted, became distended and again filled the zona. Denser fluids produced an opposite effect, the yelk became considerably contracted.

§ 102. Collating the results of his own and Barry's examinations of ova found in the first half of the Fallopian tube, Bischoff makes the following observations (p. 54:) "I cannot agree with Barry that the yelk, or as he calls it, the 'substance by which the germinal vesicle is surrounded,' of the fecundated ovum in the Fallopian tube, is composed of cells of any kind such as he has delineated in his third series, figs. 185, 188, 189, 190, 193, 194, 195, 199, 200, &c. It will I hope be allowed, that such an appearance is too remarkable to have escaped me with the use of good instruments. Moreover I can as little admit that, as Barry affirms, the substance in question which is considered by me yelk, gradually disappears by the solution of the cells forming it. The mass which in the preceding observations fills the interior of the ovum, corresponds in its appearance and condition so completely with that filling the unfecundated ovum, that it is impossible not to consider both as identical. The only difference by which, as I think, even Barry was led to his conclusion, is, that this mass no longer fills the whole interior of the zona, but is often considerably shrunk. How much soever this state is to be attended to, seeing that it probably stands in close connexion with after-changes, it gives little warrant for the supposition that a partial solution of this mass occurs. The increasing consistence of the yelk, and the reactions it exhibits with fluids, prove that the diminution of its size is owing merely to a closer aggregation of its particles, occasioned probably by the action of the fluids secreted by the Fallopian tubes.* The addition of water, as above mentioned, generally soon brings the yelk back to its former size." Bischoff considers as altogether erroneous the supposition of Barry, that the two granules or vesicles, which are often seen at this stage of development in the space within the vitellary membrane (zona) left by the contraction of the yelk, are a remains of the dissolved yelk-mass. He is rather inclined to view them as of great importance for the farther preparatory change of the yelk-mass. This view of Bischoff will be stated below. Farther Bischoff particularly observes, that it is impossible for him to coincide with Barry in his statements regarding the germinal vesicle. With the greatest care and attention, and in his last observations when he had become acquainted with Barry's paper and figures, he never could observe in the rabbit's ova from the tubes a germinal vesicle at all, and still less one enlarged and filled with cells. Even Barry's own delineations, figs. 187 and 193 *c*, Bischoff says are so uncertain and indistinct, that he is astonished at his assertions. Bischoff has indeed several times remarked in the yelk a bright spot such as Barry mentions, on the existence

* The seminal fluid ?

of which he says undoubtedly great weight is to be laid, but he does not believe it to be produced by the unaltered germinal vesicle of the fecundated ovum.

§ 103. *The process of division and subdivision of the yelk* now claims consideration. In his communication of 1838 Bischoff observes, "I have occasionally observed such remarkable forms that I asked myself the question, whether the vitellus of the mammiferous animal's ovum underwent changes of form similar to those of the ovum of fishes and batrachia?" (Willis's Wagner, p. 140.) At page 152 he conjectures that these alterations in the form of the yelk are due to this: that all the yelk-granules are at first inclosed in two, then in four, then in eight cells, and so on until each at length comes to have its own cell, which coalescing with one another, the blastoderma is produced as a preliminary to the appearance of the embryo.

§ 104. Dr. Barry's observations in his 2d series, 1839, are as follows: In the centre of the fluid, which, as above seen, Barry in his 2d series designates yelk, "there arise (this quotation is from the summary, p. 351) several (at first two vesicles, then four or more, and so on) very large and exceedingly transparent vesicles. These disappear and are succeeded by a smaller and more numerous set. Several sets thus successively come into view, the vesicles of each succeeding set being more numerous and smaller than the last, until a mulberry-like structure has been produced, which occupies the centre of the ovum. Each of the vesicles of which the surface of the mulberry-like structure is composed, contains a colourless and pellucid nucleus; and each nucleus presents a nucleolus. This mulberry-like body is found to contain a cell larger than the rest, elliptical in form, and having in its centre a thick-walled hollow sphere, which is the nucleus of this cell. This nucleus is the rudimental embryo." In § 177 of his paper itself, in § 307 of the first postscript, and in § 318 of the second postscript, Dr. Barry remarks on the resemblance between the process of division and subdivision he had just described in the mammiferous ovum, and the divisions and subdivisions first noticed by Prevost and Dumas in the yelk of the ovum of the frog, and now known to occur in the ova of various other animals.

§ 105. In his first P.S. § 307, Barry remarks that the process of subdivision into smaller and more numerous vesicles suggests the idea, originally that of Schwann, that in the interior of each vesicle there arise two or more infant vesicles, the parent vesicle in each instance disappearing by liquefaction. In his second postscript, headed "Process by which the primitive ovum undergoes division and subdivision—Probable analogy in this respect between the mammiferous ovum and the ovum of batrachian reptiles and certain fishes—The so-called yelk-ball in the mammiferous ovum compared with the 'Discus vitellinus' in the ova of other animals—Resemblance between the fecundated ovum of mammalia and that of certain plants"—Barry says that in ova which he had since examined he had found, what he suggests as likely in his first postscript to be really the case, viz., "that in the interior of each vesicle there arise two or more infant vesicles, the parent vesicle in each instance disappearing by liquefaction."

§ 106. In his third series Dr. Barry says that each of the twin cells, which according to him as above seen succeed the germinal vesicle, presents a nucleus; which having, like the germinal spot in reference to its vesicle, first passed to the centre of its cell, resolves itself into cells in the manner above described. By this means the twin cells in their turn become filled with other cells, but only two of these in each cell is destined to continue, the others as well as the membrane of each parent-cell disappear by liquefaction. There are thus now four cells. Each of these four, in its turn a parent-cell, gives origin to two; by which the whole number is increased to eight. This mode of augmentation continues until the germ consists of a mulberry-like object, the cells of which are so numerous as not to admit of being counted. Together with a doubling of the number of the cells, there occurs a diminution of their size.

§ 107. These facts, Dr. Barry says, do not confirm Bischoff's conjecture referred to in his (Dr. Barry's) second series, viz., "that in the first place all the yelk granules are inclosed in two, then in four, then in eight, &c. cells." (Willis's translation of Wagner, p. 153.) But they strengthen, he thinks, the analogy pointed out in that memoir between the early changes in the ovum of the mammifera and the divisions previously known to occur in the ovum of batrachian reptiles and some other animals; and he says it is almost superfluous to add, that he supposes the process in all to be essentially the same, and that it is in operation in other animals. Barry says that he believes it has hitherto been usual to regard the round white spot or cicatricula in the yelk of the bird's laid egg as an altered state of the discus vitellinus in the unfecundated ovarian ovum. So far from thinking that such is the case, Barry ventures to believe that the whole substance of the cicatricula in the laid egg of the bird has its origin within the germinal vesicle in the same manner as in the ovum of the mammalia, and therefore that the cicatricula in the bird's laid egg may properly be called the germ.

§ 108. Bischoff first observed the process of division and subdivision of the yelk in the ovum of the dog, but he finds that it can be more easily traced in the rabbit's ovum in consequence of its less density.

§ 109. Regarding Barry's account of the process, Bischoff remarks, "it will be admitted by every unprejudiced person that Barry did not in his second series express himself clearly on the subject; and has in his third series, as I am convinced, interpreted it quite erroneously. As above seen, Barry alleges that the yelk becomes entirely dissolved, and that the whole phenomenon, hitherto considered as a division and subdivision of the yelk, consists merely in a progressive development of cells from the germinal vesicle; from which, first two, then from these four, and from these eight, and so on, are alleged to be developed, and that not in a simple but in a very complicated manner.

"I have above taken from these allegations their grounds of support, by disproving the solution of the yelk and the persistence and metamorphosis of the germinal vesicle, before the process in question begins. I think it is straining things very far not to recognize in the globules filling the interior of the ovum during the period in question, exactly the same elements as those of which the contents of the vitellary membrane (zona) consist, as well before as immediately after fecundation." In the analogous phenomenon in other animals, Bischoff goes on to say that "it cannot for a moment be admitted that the yelk dissolves and that a new substance proceeding from the germinal vesicle produces those globules. Athough there be some differences in the mode of division and subdivision of the yelk in different animals still the process itself is in all essentially the same, and consists in a progressive resolution of the yelk into smaller and smaller globular segments. No other observer has attributed any direct part in the process to the germinal vesicle, but all agree that this itself has already disappeared when the subdivision commences."

§ 110. Careful examination of the globular masses of yelk themselves also contradicts Barry's view of their condition and mode of formation. There is, indeed, to be observed inclosed in each of them a particular central mass, a clear speck, a nucleus. But this does not at all resemble those forms described and so distinctly delineated by Barry. Bischoff has never been able to perceive in any of those globular masses of yelk the bright shining central point and the progeny of cells proceeding from it, as alleged by Barry. Lastly, he has never been able to discover, notwithstanding the greatest attention, any trace of that large elliptical cell and its nucleus with transparent centre and granulous periphery, which Barry says he has observed in ova at the end of the tube among the other globular masses of yelk, and holds to be the first trace of the embryo.

§ 111. Bergmann, Reichert, Vogt, Bagge, and more recently Kölliker, have occupied themselves with this remarkable process of division and subdivision of the yelk, but not in the mammifera. From the similarity of the process in almost all animals, however, their researches may be very advantageously applied to assist the inquiry into the nature of the process in the mammifera. They will be found to give a probable explanation of the observations on which Dr. Barry has founded his inaccurate and disjointed views and delineations.

§ 112. From his observations on the ova of the frog, Bergmann (Müller's Archiv, 1841) considered the first stage of the process as a mere division of the yelk, and that the segments are not inclosed in a particular investment or cell-membrane; and that therefore they cannot be called cells. In the later stages, however, he satisfied himself that the segments are cells, i. e. they possess a very delicate investing membrane, by which the yelk-granules are held together. He then remarked also in each of these cells a clear round spot, which became distinct on slight pressure of the cell. From this he is of opinion that the cell-membrane is formed around a previously-existing globular mass, which then presents itself as cell-contents. As to the clear spots observable in these cells, Bergmann is doubtful whether or not they are to be viewed as nuclei. On the whole Bergmann considers the subdivision of the yelk as leading to the formation of cells.

§ 113. The result of Reichert's researches (Müller's Archiv, 1841) on the point is, that all the parts or globular masses which come into view during the process of subdivision, nay, even the whole as yet undivided yelk-ball as well as the elements which afterwards come together for the formation of the embryo, are surrounded by a proper fine investment, and are therefore cells. All these cells are from the very commencement of the process of division inclosed within one another, the smaller which appear later within the larger and lastly the two which first appear within the cell, forming the whole yelk. The process of division consists only in this, that the inclosed pre-existing cells become free when the inclosing mother-cells dissolve. In this manner the first two yelk-cells appear, when the mother-cell inclosing them and investing the whole yelk dissolves. When the two cells, thus become free, dissolve in their turn, the four next contained within them become free, and so on.

§ 114. According to Vogt* the process of division of the yelk in Alytes obstetricans is not only confined to one half of the ovum, which is also the case in some other animals, but he alleges that there is no division of the yelk-mass through and through, that the division affects the surface only, and is at last brought about internally by a folding in of the vitellary membrane. This process of grooving he alleges stands in no direct relation to the formation of the cells which afterwards appear and serve for the development of the embryo, seeing that this formation of cells begins only when the grooving process is finished and the yelk has again become smooth.

The multiple germinal spots of the germinal vesicle, Vogt considers to be cells, which are inclosed within the germinal vesicle as within a mother-cell. Vogt, like all preceding observers, satisfied himself that the germinal vesicle, which he had shortly before readily found and observed, has always disappeared after the egg is laid. Now, however, he succeeded in again finding the germinal spot-cells dispersed in the cortical layer of the yelk, which, it would appear, had become free by the solution of the germinal-vesicle cell. These germinal-spot cells he afterwards observed in the cells arising and arisen after the grooving process, in which they appear to play the part of a nucleus; and although he does not on that account connect the grooving of the yelk with this formation of cells, he yet thinks he has proved that the latter takes place in consequence of the germinal-spot cells, each becoming surrounded by a group of yelk-granules, and these by a cell-membrane, so that cells are formed around cells. Vogt, however, at the same time admits that, in addition to the process just mentioned, cells form in the centre of the yelk, and this without the concurrence of these germinal-spot cells, or any newly-formed ones similar to them, by individual masses of yelk-granules becoming directly surrounded by cell-membranes without the concurrence of a nucleus.

§ 115. Bagge's† researches were made on the ova of Strongylus auricularis, and Ascaris acuminata. In the ova of these two viviparous entozoa the process of division of the yelk takes place, and here also ends in the formation of the cells which immediately compose the body of the embryo. In the ova of these animals also the germinal vesicle disappears after fecundation. But then there appears in the middle of the as yet undivided yelk a small clear cell. This becomes somewhat elongated, contracts in the middle, and assumes a fiddle-shape.

* Untersuchungen über die Entwickelungsgeschichte der Geburtshülfer Kröte. — Solothurn, 1841.

† Diss. de evolutione Strongyli auricularis et Ascaridis acuminatæ.—Erlangæ, 1841.

At last it is divided in the middle, and there arise out of it two vesicles which withdraw themselves towards the two poles of the somewhat oval yelk. The division of the latter now begins, so that each half incloses one of the two vesicles. As soon as this has taken place, the same process is repeated with the vesicle in each half. These also divide, and the repeated division of the yelk follows this division, and each part of the yelk thus always contains a small vesicle inclosed within it.

§ 116. In regard to the nature of the process in the rabbit, Bischoff expresses himself to the following effect (p. 75):—"I have already above expressed my conviction that the germinal vesicle collapses or dissolves when the ovum leaves the ovary, although the period at which this takes place does not appear to be quite definite; nay, I even hold it for possible, that the germinal vesicle itself sometimes does not disappear until the ovum is in the tube: I have however never seen it in an ovum in the tube. Instead of it, I then sometimes saw in the interior of the yelk, a clear somewhat smaller spot than is formed by the germinal vesicle. Hitherto, however, I have in vain endeavoured to obtain a more distinct view of this clear spot, and that in a separate state; still I have satisfied myself that it is not the germinal vesicle itself. In this stage there appear the two granules or vesicles above mentioned at the surface of the yelk, to which I cannot refrain from ascribing some definite and important use, as they are found also in the ovum of the dog at this stage. Hereupon the division of the yelk commences, and each successive segment has in its interior a similar clear spot like that which the whole yelk-ball shortly before appeared to contain. It is observable only under particularly favorable circumstances, and even when I succeeded in getting the best view of it, it was impossible for me to come to any certain conclusion regarding its nature. It is distinguishable by its bright shining transparent appearance, and its boundary is determined more by the yelk-granules surrounding it, than by any proper membrane. I could never recognize in these clear central parts of the globular segments of the yelk, either a cell-membrane or a nucleus, but the whole appearance corresponded most with that of an oil-globule, around which the yelk-granules had been collected. In this respect these clear points agree entirely with those which are also observed in the globular segments of the yelk of the frog's ovum, and which, as we above saw, Vogt considers as the germinal spots of the germinal vesicle and to be themselves cells; Bergmann is doubtful whether they should be called nuclei or cells; and Reichert does call them nuclei. Were I called on to choose from among these designations, I would give the preference to the last, because in fact cell-nuclei occur in other places, which are extremely clear and transparent, and because I believe that they are actually products or descendents of a cell-nucleus, viz. the germinal spot. I would however avoid using the term nucleus for those clear central parts of the globular segments of yelk, because it is usual forthwith to associate it with a cell, which I cannot recognize the globular mass of yelk to be.

§ 117. "I will therefore rather describe than name, and suspend any opinion as to the nature of the parts in question, until farther observations give greater certainty. I believe that the clear spots are descendants of the germinal spot of the germinal vesicle. When the germinal vesicle has dissolved, the germinal spot appears, probably in consequence of the action of the male semen, to become enlarged and converted into a clear body like an oil-globule, to become in fact more like a vesicle. Whether the germinal spot so changed again passes from the periphery of the yelk, where it must be after the solution of the germinal vesicle, to the centre, here to divide, and the two parts again return to the surface, or what is more simple and probable, the germinal spot remaining at the surface of the yelk divides into two parts, I cannot say, because my observations are not sufficiently complete, and because both opinions are possible."

§ 118. However that may be, Bischoff considers the two granules or vesicles above referred to, as being observed swimming in the fluid filling the space between the zona and the shrunk yelk before the process of division and subdivision commences, as the two parts and descendants of the germinal spot, around which the yelk-grains collect in two groups, by which the first division of the whole mass is produced. In the dog especially he thought he perceived that those two granules at the surface of the yelk-mass consisted of such a clear corpuscle, as are remarked in the subsequently formed globular masses of yelk which were beset with a single layer of yelk-granules. By the continued accumulation of yelk-granules around them, the previously single yelk-mass becomes divided into two parts. Then there probably takes place in each of the clear central corpuscles, a new division, followed by a new grouping of the yelk-grains around, so that now there are in the whole four parts, from these four arise eight in the same way, and so on.

§ 119. For the reasons he adduces, Bischoff cannot, as he formerly did, consider as *cells* those globular segments arising from the division and subdivision of the yelk of the rabbit's ovum during its passage through the Fallopian tube, nor recognize in the whole process one of cell-development, (p. 79.) He considers it a process *sui generis*, which, as the result shows, appears to be a preparation for the development of true cells. Conglomerations of elementary granules, he continues, such as the globular masses of yelk here represent, are by no means uncommon. They are to be found in pus and plastic exudations, and here constitute Gluge's inflammation globules; in milk they form colostrum-corpuscles.

§ 120. Kölliker* has carefully followed the first processes in the fecundated ovum of several invertebrata. He finds (p. 84) that the germinal vesicle and its spot disappear after fecundation, but that in the centre of the yelk there is developed a nucleated cell which he calls *embryo-cell.* Within this cell are formed two young ones, which become free by the solution of their mother-cell. Each of these two cells become surrounded by one half of the yelk-mass. This constitutes the commencement of the division of the yelk. Within each of the two cells two younger ones are developed, which on becoming free by solution of their parent-cell, become surrounded by a subdivision of the yelk-mass. And so on the process goes, the cells of each succeeding brood drawing around them the yelk substance, until this is all consumed in the process, and there remains in its stead the mulberry-like body consisting of an agglomeration of cells, the descendants of the first embryo-cell. From this it appears that Kölliker agrees with Bagge, that the embryo-cell formation always precedes the process of division, and that the latter is in some manner caused by the former. He does not however agree with Bagge that the cells multiply by fissiparous generation, but believes that they increase by endogenous generation as above mentioned, and by the nucleus, first discovered by himself, of each of the successive embryo-cells, as in its turn it becomes parent-cell, dividing into two parts, around each of which a cell-membrane forms.

§ 121. The process of division of the yelk does not take place in Ascaris dentata, Oxyuris ambigua, Cucullanus elegans, Bothryocephalus and Distoma, and is therefore not to be considered essential. The development of embryo-cells, however, appears to occur in all, and is consequently to be considered the essential part of the process. Kölliker is the discoverer of this development of embryo-cells without division of the yelk. Partial division had been previously observed by Vogt in Alytes obstetricans. If, as above hinted at as probable, a division takes place in the bird's egg, it must be still more partial.

§ 122. From his observation of the first processes in the ovum of the invertebrata after fecundation, Kölliker is disposed to interpret the appearances presented by the rabbit's ovum as described by Bischoff, in the following manner: After fecundation the germinal vesicle and spot disappear. In the centre of the yelk, the first embryo-cell makes its appearance, as the clear speck described by Bischoff as of smaller size than the germinal vesicle, and which he considered might be the germinal spot enlarged. Around this first embryo-cell, the loose yelk closely contracts. Within this embryo-cell two new ones arise from its divided nucleus—for Kölliker (p. 119) thinks a nucleus exists, though not observed by Bischoff on account of the difficult nature of the investigation—and become free by solution of the mother cell. When this takes place, these two new cells become

* Beiträge zur Entwickelungsgeschichte wirbelloser Thiere. In Müller's Archiv, Nos. i. and ii. for 1843.

each surrounded by a mass of yelk. The previously single yelk-ball is thus divided into two. And so proceeds the progressive formation of embryo-cells by endogenous generation, attended by the division and subdivision of the yelk into segments to inclose the new embryo-cells which have become free, until the process is completed.

§ 123. Kölliker does not agree with Bischoff in the opinion that the two bodies observed swimming in the fluid which accumulates in the space between the vitellary membrane, and the shrunk yelk are the nuclei, around which, in the process of division of the yelk, the two first segments are collected, but he thinks they are simply the remains of the germinal spot dissolving.

§ 124. Speaking of the results of Barry's researches on this subject, Bischoff (p. 68, 71) says he is sorry to have to consider them as the effect of fancy and enthusiasm for the cell-theory; and Kölliker does not hesitate to apply to them the epithet *fabulous*. Dr. Barry, however, must surely have seen some of the realities at least, that are appreciable, though he has misinterpreted them in so extraordinary a manner. Supposing the yelk to have become fluid, he appears to have mistaken the real yelk-ball for the germinal vesicle enlarged and rendered opaque by being filled with cells. Then finding the two cells derived from the first embryo-cell, he has put them down as those which alone, out of the great number he supposed to be formed within the germinal vesicle, come to perfection. The yelk-granules he appears to have mistaken for those which he supposes do not come to perfection. And so on runs his mistake, which it will be perceived is traceable to his conceit regarding the fate of the germinal vesicle.

§ 125. The following table is given by Kölliker, to show the relation of the yelk to the development of the embryo-cells in different animals:

| I.<br>*Embryo-cells developed free in the Yelk.*<br>(No subdivision of the Yelk.) | | II.<br>*Embryo-cells surrounded by the Yelk.*<br>(Subdivision of the Yelk.) | |
|---|---|---|---|
| 1. The first embryo-cells are small, and assimilate the yelk slowly. | 2. The first embryo-cells are large and immediately take up the whole yelk. | 1. The first embryo-cells surround themselves with a part of the yelk only.<br>(*Partial subdivision.*) | 2. The first embryo-cells surround themselves with the whole yelk.<br>(*Complete subdivision*) |
| *a. Yelk granulous.*<br>Bothryocephalus.<br>Tænia.<br>Distoma tereticolle. | | *a. The last globular masses of yelk become cells.* (?)<br>Coregonus palaea.<br>Alytes obstetricans. | *a. The last globular masses of yelk become cells.* (?)<br>Rana.<br>Triton.<br>Lepus cuniculus.<br>Canis familiaris. |
| *b. Yelk fluid.*<br>Ascaris dentata.<br>Oxyuris ambigua. | *b. Yelk fluid.*<br>Cucullanus elegans. | *b. The last globular masses of yelk never become cells.* (?)<br>Sepia vulgaris.<br>Loligo sagittata. | *b. The last globular masses of yelk never become cells.* (?)<br>Ascarides permultæ.<br>Strongyli nonnulli.<br>Nereis.<br>Botryllus.<br>Ascidiæ compositæ<br>Pycnogonum.<br>Acolidiæ. |

§ 126. In four ova found in the middle of the Fallopian tube of a rabbit, around which the gelatinous-looking investment was still very thin, in which the division and subdivision of the yelk had not commenced, but in which the yelk-ball was a coherent round mass, not filling the interior of the vitellary membrane (zona), but suspended in a transparent fluid, which, (together with two small yellow shining grains or cells in three of the ova), constituted the rest of its contents,

Bischoff observed a very remarkable phenomenon, viz., a rotation of the yelk-ball, produced by cilia on its surface, within the zona. The direction of the rotation was from the uterus towards the ovary. The motion was continuous, but after adding some aqueous humour to prevent desiccation, it ceased.

§ 127. Bischoff has not hitherto made a second observation of the same kind. He has observed no such appearance in the ovum of the dog, though examined under circumstances when it might have been expected. In ova of the rabbit farther developed, he has not observed the rotatory motion. His examination of ova at earlier periods were made before he was aware of the phenomenon. He expresses himself however quite convinced of the correctness of the observation above mentioned, and that there is the less reason to suppose there could have been any error, as similar rotatory phenomena are known to occur in the ova of other animals. It is to be remembered, however, that as Reichert observes, the rotation of the yelk in the ova of other animals does not occur until *after the process of division and subdivision*, by which the yelk is resolved into cells. It is with the cells on the surface that the vibratile cilia are connected.

§ 128. Dr. Barry mentions in his second series having observed rotatory motions of a mulberry-like object in vesicles under the mucous membrane of the uterus, but this can have nothing in common with Bischoff's observation. Barry is as decided in opinion that the object he examined *was not*, as Bischoff is that the object he examined *was*, an ovum.

§ 129. *Correlations of the changes in the ovum above examined.* Dr. Barry in his 2d series describes eight different stages of development as being presented by the ova of the rabbit in their passage along the Fallopian tube. All this, and his numerous subsequent stages in the uterus, Bischoff says are quite unnecessary, and it is to be observed that Dr. Barry himself in his 3d series, speaks of his subdivision into stages, as having been adopted for a "temporary purpose."

§ 130. The ova on entering the Fallopian tube are still surrounded by the cells of the "discus," and *membrana granulosa*, but these cells, according to Bischoff, have lost the fusiform shape they had assumed before leaving the ovary, and again become round; and it is very soon remarked of them that they are in process of solution, whereby, says Bischoff, their sharp outline is vanished, and they appear again to be fused together.

§ 131. Somewhat further on in the tube, Bischoff says, these cells have almost entirely disappeared; some remains of them only are observed on the vitellary membrane (zona), and at last these also disappear entirely, so that the ovum now appears naked, and the vitellary membrane (zona), is seen to be immediately beset with spermatozoa, but which Bischoff never saw still in activity. At this stage the yelk has the same appearance as in the mature ovarian ovum, but it soon shrinks and no longer fills the cavity of the zona. Bischoff never found a germinal vesicle in such ova.

§ 132. Barry observed the first appearance of the gelatinous-looking investment around ova, found at about an inch from the infundibulum in the Fallopian tube, and describes it as being already very evident in ova from the middle of the tube. Bischoff appears not to have observed the appearance of the investment in question, until the ova were in the middle of the tube; at the same time that he saw the rotatory movements of the yelk.

§ 133. On entering the second half of the Fallopian tubes, the process of division and subdivision of the yelk commences and goes on together with the thickening of the gelatinous-looking investment as it passes on towards the uterus.

§ 134. The process of resolution of the yelk into smaller and smaller globular masses going on and the ovum surrounded by a thick gelatinous-looking investment, it passes from the tube into the uterus. This occurs at the end of the third or beginning of the fourth day *post coitum.* So that the period of the passage of the ova in the rabbit through the Fallopian tubes falls between the eleventh and seventy-seventh hours.

§ 135. It appears that the ova pass through the first part of the tube more quickly, more slowly along the latter part. As to the means by which the ovum is carried along: the vibratile cilia of the epithelium of the Fallopian tube appear to be one means, and it is to be remarked, in connexion with the more rapid passage of the ova through the first part of the tube, that here the cilia are most active. But the principal means, Bischoff thinks, are the contractions of the Fallopian tubes themselves; he has seen such contractions very evidently. It is to be remarked that the contractions are in a direction opposite to that by which the conveyance of the seminal fluid towards the ovaries was stated to be effected; but this difference in the direction of the contractions of the same part, Bischoff remarks, is not singular; contractions in opposite directions take place naturally, e. g. in the gullet of ruminants.

§ 136. It has been already seen that in the dog, Bischoff says, no albuminous investment is added to the ovum in its passage through the tube. Some remains of the granules or cells of the discus which surround the ovum when it enters the tube, and which have again become round, though almost dissolved and vanished, continue attached to the ovum when it enters the uterus, and indeed appear to have become united with the external surface of the zona, which therefore presents an uneven and slightly granular appearance. Might not this be looked upon as a trace analogous to the albuminous layer?

§ 137. The yelk undergoes the same process of subdivision as above described in the rabbit.

§ 138. The ova in the dog are received into the Fallopian tubes at a later date *post coitum* than in the rabbit, and they pass along the tubes more slowly, so that they are not found in the uterus until about the eighth day, though this is said to be different in different individuals.

§ 139. Of the human ovum in, and its passage through the Fallopian tube nothing is known.

### IV. The development of the ovum in the uterus until the first appearance of the embryo.

§ 140. Of the two vesicles of which Baer found the ovum of the dog in the uterus to consist, he considered the outer the same as the outer membrane of the ovarian ovum, and now called it *membrana corticalis or chorion*, as he believed he had observed the development of villi upon it. The inner vesicle he considered as resulting from the fusion of the yelk-grains during development, and called it first *membrana vitelli;* and a dark spot observed on one side of it he first considered as the analogue of the *blastoderma* of the bird's egg. (Epistola, pp. 10 and 23.) He afterwards changed his opinion in so far that he considered the whole inner vesicle as blastoderma, which from the very commencement presents itself in the form of a vesicle. (Heusinger's Zeitschrift, ii. p. 174; Entwickelungs-geschichte, ii. p. 184.) The dark spot in it which he saw to be at first round, afterwards elongated, he recognized as the place where the embryo is developed from a primitive streak in exactly the same way as the embryo of the bird. (Entwickelungs-geschichte, ii. p. 189.) He says further, though in a very brief and unsatisfactory manner, that the blastoderma splits into two layers, an animal and a vegetative, as in the bird's egg, and grounds thereupon his whole account of the development of the embryo. (Entwickelungs-geschichte, ii. p. 192, r. u. 208-z.) But it does not appear certain whether this statement was the result of direct observation or only a conclusion drawn from the analogy between the ovum of the bird and the mammal.

§ 141. Coste also saw and described the ovum, first as a transparent vesicle, consisting of two membranes, the one inclosed within the other. The inner membrane he at first supposed to be the germinal vesicle enlarged, but afterwards repudiated the opinion. In his 'Embryogenie' he considered the outer membrane of the uterine ovum as the vitellary membrane (zona) of the ovarian ovum unchanged except that it was increased in size. He therefore still called it in the uterus vitellary membrane. The inner he considered as a product of development and called it blastoderma. At the seventh day he observed on the latter a spot, already seen by Cruikshank on the sixth, and called it "tache embryonnaire," because from it the development of the embryo proceeds. The blastoderma, he says, must be considered as composed of two principal layers; an external and an internal, and an accessory layer enveloping the latter. In the rabbit's ovum of the seventh day we may, he says, but not without much difficulty, succeed in demonstrating what will be afterwards still more evident, that the "tache embryonnaire" may be separated into two concentric layers, which may be traced into almost the whole extent of the blastoderma, which is consequently also formed of two layers. The "tache embryonnaire" is at first round, then elliptical, then guitar-shaped, soon after this the head and tail ends of the embryo may be recognized.

§ 142. It is easily seen, however, Bischoff observes, that there is an important hiatus between the stage of development, at which the ovum is at the end of the Fallopian tube and the condition in which it is here represented. Dr. Barry supplies observations referring to the period of this hiatus when the ova are from one fifth to one half of a Paris line in diameter.

§ 143. In the uterus, according to Dr. Barry, a layer of vesicles of the same kind as those constituting the mulberry-like object (the yelk in its last and most minute state of subdivision or rather, as above shown to be probable, the agglomeration of the last descendants of the first embryo-cell) makes its appearance, and, like an epithelium, lines the whole inner surface of the membrane, which now incloses the yelk—the thick transparent membrane or zona pellucida (vitellary membrane). The mulberry-like structure then passes from the *centre* of the yelk to a certain part of the layer just described (the vesicles of it coalescing with those of the mulberry-like structure, where the two sets are in contact, to form a membrane; Dr. Barry says, the so-called germinal membrane, or what has been denominated its "serous lamina" by authors on the ovum of the bird, but really, according to him, the future *amnion*). The interior of the mulberry-like structure is now seen to be occupied by a large vesicle containing a fluid and dark granules. In the centre of the fluid of this vesicle is a spherical body composed of a substance having a finely-granulous appearance, and containing a cavity filled with a colourless and pellucid fluid. This hollow spherical body seems to be the true germ. The vesicle containing it disappears, and in its place is seen an elliptical depression (*area pellucida* of authors on the bird) filled with a pellucid fluid. In the centre of this depression is the germ, still presenting the appearance of a hollow sphere. In regard to this, his account of the true germ in his 2d series, Dr. Barry remarks in a note that perhaps it would be more correct to consider the vesicle itself (which forms the interior of the mulberry-like structure) with the whole of its contents as the true germ. In his 3d series, Dr. Barry substitutes the term "rudimental embryo" for "germ." This latter name he now applies to the "twin-cells, foundation of the new being, group of cells, mulberry-like object," which, as has been seen, he alleges is formed out of the germinal vesicle. Thus, according to Dr. Barry, the rudimental embryo is the nucleus of a cell. It passes from a spherical into a linear form; in some stage of which latter state it appears to correspond to the "primitive trace" of authors on the ovum of the bird. This alleged transformation of a nucleus into the embryo appears to Dr. Barry to be a process similar to what is in operation in other instances where the product does not extend beyond the interior of a minute and transitory cell. Making allowance, indeed, he says, for a difference in form and size, the description given of the mode of production of the one might be applied to the other.

§ 144. In the recapitulation of his 2d series Dr. Barry continues; the germ (i. e. the rudimental embryo of his 3d series) separates into a central and a peripheral portion, both of which at first appearing granulous, are subsequently found to consist of vesicles. The central portion of the germ (i. e. rudimental embryo) occupies the situation of the future brain, and soon presents a pointed process. This process becomes a hollow tube, exhibiting an enlargement at its caudal extremity, which indicates the situation of the future sinus rhomboidalis. Up to a certain period new layers come into view in the interior of the central portion of the germ (i. e. rudimental embryo), parts previously seen being pushed farther out.

§ 145. From the region occupied by the germ there arises a hollow process, which by enlargement is made to line the inner surface of the ovum; that is to say, the inner surface of the membrane entering into the formation of the amnion, (which corresponds to the "serous lamina" of authors,) and the process now lining it represents an incipient state of the subsequently "vascular lamina" of the umbilical vesicle; a lamina continuous with the structure corresponding to the "area vasculosa" of authors on the evolution of the bird.

§ 146. There does not occur in the mammiferous ovum any such phenomenon as the splitting of a "germinal membrane," into the so-called "serous, vascular and mucous laminæ." Nor is there any structure entitled to be denominated the "germinal membrane;" for it is not a previously existing membrane which originates the germ (rudimental embryo); but it is the previously existing germ (rudimental embryo), which by means of a hollow process originates a structure having the appearance of a membrane.

§ 147. Bischoff affirms that in most of these statements Barry is in error, and in particular calls in question all that he says of the first traces of the germ and embryo, and the want of analogy with what is known of the bird's egg. Had Barry, Bischoff remarks, extended his observations to more advanced ova than he has done, he must necessarily have convinced himself of the erroneous interpretations he has given to many particulars otherwise correctly enough observed.

§ 148. Bischoff (p. 85) observes that at the stage under consideration the changes succeed each other so quickly, and the difficulty in finding and handling the ova are so great, that he should scarcely have had a sufficient series of observations in the number (70) which he examined, had he not hit upon the expedient of making use of the ova contained in the two horns of the uterus of one and the same animal for separate successive examinations. When he expected the ova in the uterus at a certain stage, he cut out from the living animal the one horn of the uterus after ligature of the mesometrium and uterus above and below, an operation performed in a few minutes without any loss of blood, and without any considerable suffering to the animal. He then examined the ova, and determined from their condition when to examine the other uterus, with or without killing the animal. As the ova are generally equally developed in the two sides of the uterus at the same period, he obtained in this manner a certainty regarding the succession of the appearances, which was not to have been obtained even by the sacrifice of a much greater number of animals.

§ 149. The ovum, high up in the uterus, has at first the same size and appearance as in the lower end of the tube, and is readily found on account of the glancing of its albuminous layer, although indeed with much more difficulty in the wide uterus than in the tube. In ova just taken out of the upper part of the uterus and examined without any addition, the yelk is seen to be no longer of a mulberry appearance, but uniform and finely granular on the surface, and quite filling the vitellary membrane (zona), so that it would be exactly similar to the yelk of an ovarian ovum, were it not much paler and more transparent. But if any fluid be applied it is observed for the most part, that after some time the yelk mass contracts, and by and by its former mulberry appearance again comes into view, quite distinctly and sharply defined.

§ 150. At the next stage, the ova are, on the whole, still very like the preceding. The albuminous layer is still present, likewise the vitellary membrane (zona), and the size of the ova is nearly the same. But instead of the uniform appearance of the interior of the ovum, there are now remarked, when the focus of the microscope is adapted to its surface, cells distinctly pentagonal or hexagonal, joined to each other so as to form a layer, which is applied against the inner surface of the zona. The cells are filled with pale, finely-granular contents, and possess a very clear nucleus, not visible in the recent state. When the focus of the microscope is adapted to a deeper part of the object, it is observed that the ovum now represents in its interior a globular hollow filled with a clear fluid, and that the cells composing the layer above mentioned, as now lining the vitellary membrane (zona), whilst they are pressed flat against it, project towards the interior in a round form. At some one place there lies a dark heap of globules which are quite similar to, and evidently identical with those resulting from the previous division of the yelk.

§ 151. In this condition the ova grow, whilst they proceed farther down in the uterus, pretty rapidly, but in an especial manner, the vitellary membrane (zona), and along with it the layer of cells, which is closely applied to it, becomes more distended By this the albuminous layer is rendered thinner. At last it and the vitellary membrane (zona) coalesce entirely with each other, and now as one common, still more or less thick, layer, constitute the outer investment of the ovum. The layer of cells at the inner surface of this investment is very distinct; the individual pentagonal or hexagonal cells, with their nuclei which are pressed against each other, are easily distinguished; and they unite together more and more in the form of a membrane, and begin to form a very delicate internal vesicle, which lies close to the external investment, but which, after some time and after contact with a fluid, begins to separate from it. At one place the dark accumulation of yelk globular masses is still seen lying, but it diminishes more and more.

§ 152. Barry has delineated ova of this kind in his second series, figs. 111, 112, 113, 114, 115, 116, and in his third series, fig. 234. He has also correctly recognized the membraniform layer of cells at the inner surface of the zona, and the heap of globular masses of yelk. He calls the membraniform layer, *amnion*; which, however, Bischoff observes cannot yet be spoken of as existing, although this membrane composed of cells at a later period contributes a part for the formation of the amnion. Barry says nothing farther of the heap of yelk-globules; but he asserts that in it he has still seen that large elliptical vesicle with that clear centre (true germ of his 2d series, rudimental embryo of his 3d series) from which he alleges the embryo is produced, but of which Bischoff as well as several other observers to whom he showed such ova, could never observe the slightest trace.

§ 153. The question now arises, how the condition of the ova here described is evolved from that in which the whole vitellary membrane (zona) was seen equally filled by the globular masses of yelk pressed closely together, and that uniform appearance of the ovum thereby produced? Bischoff considers the following to be the process (p. 89.)

§ 154. When the ova have arrived at the above-mentioned stage, the globular masses of yelk begin to be changed into cells, by becoming surrounded by a fine investment, a cell-membrane. The clear spot previously remarked in each globular mass of yelk and which, as above seen, Bischoff thinks is a descendant of the germinal vesicle, becomes the nucleus of such a cell; but the rest of the globular mass, which has evidently thinned by the absorption of fluid by which it had become clearer, becomes the fine granulous contents of this cell, in regard to which it cannot but be seen that it is identical with the elements of the yelk of the ovarian ovum.

§ 155. From the observations of Kölliker above noticed, however, it would rather appear that the cells, which at last replace the minute globular masses of yelk, are the last descendants of the embryo-cell which have taken up the last remains of the yelk granules.

§ 156. The globular masses of yelk are not all at the same time replaced by cells, but gradually, and, as it appears, those first which are in immediate contact with the inner surface of the vitellary membrane (zona). The rest form the heap which is still for some time to be seen, but by and by as the ovum grows become expended in the formation of cells, which at last at the end of this stage line the whole inner surface of the ovum as a membraniform layer. There arises in this manner a second interior vesicle in the ovum, towards the formation of which all the processes hitherto described tended; and it is this vesicle which is thus the first product of development on which the materials of the ovum were expended, and for which they were sufficient. This vesicle forms the foundation for the development of the embryo, and Bischoff therefore calls it *vesicula blastodermica*.

§ 157. This process of formation of the blastoderma from the yelk is somewhat similar to that conjectured by the author of this report, but of course irrespective of the cell-theory which had not then been broached, to take place in the bird's egg, viz. that the central matter of the bird's egg serves for the extension of the blastoderma. According to this the yelk of the mammiferous ovum would represent the matter around the germinal vesicle and also that in the centre of the yelk of the bird's egg, (*Vitellus Conformationis* of Reichert.)

§ 158. The fourth and beginning of the fifth day, *post coitum*, is the period during which the above process goes on.

There now follows, in Bischoff's observations, a small gap. The last-described ova were one fifth of a line in diameter; the next observed were one half of a line. He thinks, from the condition immediately to be mentioned, that nothing important in this interval escaped him. Still he is sorry for it, because it is in this interval that Barry's wonderful figures as he calls them, 121-126, occur, which refer to ova two fifths, one fourth, and one third of a line respectively. In their distant resemblance to the area germinativa of the bird's egg, Bischoff says they will strike many. Of importance they cannot be, he continues, as nothing of them is afterwards to be found. But there is still perhaps something present which might give some explanation. As they stand, Bischoff declares the figures and their description to be quite unintelligible to him. The figures referred to, Dr. Barry states, represent the embryo and incipient amnion. In the ova represented there had certainly been "no fixed relation between their degree of development and their size, locality, or age."

§ 159. Ova of one half line and more in diameter are those which have often been seen and described by previous observers. They appear, when just taken out of the uterus, as simple transparent vesicles: they lie quite free in the uterus. When put into a fluid (not water, if it is wished to examine them further,) it is soon seen that they are composed of two vesicles hitherto lying closely applied one within the other, which now are probably loosened from each other by the entrance of the external fluid, and become more or less separated. Both vesicles appear to the naked eye almost equally transparent. But under the microscope it is readily seen that they have a different structure. The outer is quite homogeneous, pretty strong, but so thin that it does not, even under the microscope, present when folded a double contour as an indication of its thickness. When it falls together it forms sharply-defined folds, such as distinguish the capsule of the lens. The inner membrane, on the other hand, appears quite distinctly to be composed of primary cells, pressed into a polygonal form, the edges of which applied against each other are still to be distinctly recognized as lines. When just removed from the uterus, the nuclei of these cells are not distinctly seen; but they become more and more distinct, especially after the application of some reagent. The cell-contents have a pale, finely-granular appearance; and generally the molecules surround the nucleus in a circle. All this is recognized better when the outer vesicle is carefully torn open with two sharp-pointed needles under the simple microscope and the inner one allowed to come out. This inner vesicle then presents itself as extremely delicate, soft, and, after a short maceration, readily becoming diffluent. Though he subjected this second vesicle to the most careful examination, and that in not a small number of ova, Bischoff could discover nothing farther,—no trace of the future embryo, no darker spot, no larger vesicle, none of the wonderful appearances represented in the figures of Barry.

§ 160. From what has been said, it is evident that the outer of these vesicles is produced by the union of the vitellary membrane (zona) with the albuminous-like investment; and, as the ovum enlarges, becomes at last a fine membrane. Bischoff for the present calls this vesicle the *external membrane of the ovum*. The inner vesicle is evidently that the formation of which from the yelk has been above traced, and called by Bischoff *vesicula blastodermica*. It has grown and continues to do so by the increase of the cells forming it, but how this takes place Bischoff has not been able to make out. It is possible, he says, that new cells are formed from new formative materials drawn in from the uterus.

§ 161. In the blastodermic vesicle (fifth day), an opaque spot becomes observable. The most careful examination did not enable Bischoff to ascertain more of it than that there is at the place an accumulation of cells and nuclei, by which a thickening of the blastodermic vesicle was here produced. Burdach and Baer have called this spot *cumulus germinativus:* Coste, and after him Wagner, *tache embryonnaire*. Bischoff calls it "area germinativa" (*Fruchthof*), as it is quite evident that it is in it the embryo is developed.

§ 162 Ova somewhat older resemble the preceding at first view, except that they are larger. They still lie quite free, are now easily found at different places of the uterus, and still appear as round transparent vesicles. But a closer examination showed Bischoff that an important step in development had been made in them. He found that at the *area germinativa* the blastodermic vesicle consisted of a *double layer;* a very thin stratum of delicate nucleated cells having here begun to form or to separate from it at its inner surface. This proves an important correspondence of the mammiferous ovum with that of birds, in which, as is known from Pander's researches, the germinal membrane soon after the commencement of incubation presents two layers, the upper called *serous* or *animal*, the under *mucous* or *vegetative*.

§ 163. These layers of the blastoderma in the mammiferous ovum have been denied most decidedly, as above seen, by Barry; but Bischoff expresses himself fully convinced of their existence and their importance. He therefore justly lays great weight on his observation, which is confirmatory of previous statements by Baer (part 2d of his Entwickelungs-geschichte, pp. 198 and 208,) and Coste (Embryogenie, pp. 113 and 460.) In the rabbit's ovum, then, from the time it has attained the size of from one and three quarters to two lines, two layers of the blastodermic vesicle can be demonstrated; which Bischoff therefore from this time calls *serous* or *animal* and *mucous* or *vegetative*. In the mammifera Bischoff has observed nothing like Reichert's "investing membrane;" and in the bird's egg he has not examined into the point.

§ 164. In ova about the sixth day, and about two lines in diameter, somewhat elliptical and still quite free and transparent, Bischoff observed with the naked eye, but better with a magnifying-glass, very small inequalities or processes on the surface of the external membrane of the ovum. More highly magnified under the microscope they appeared to be formed of a homogeneous transparent mass, in which numerous small molecules were imbedded. Neither cells nor nuclei were to be recognized in these processes, with or without the application of any reagent; at a later period, however, cells appear. As their progressive growth shows, these processes are nothing more than the so-called villi of the *chorion*. By this development of villi upon it, the external membrane of the ovum is proved to belong to the chorion; although, as will be afterwards shown, it does not yet, Bischoff thinks, alone represent that membrane.

§ 165. Barry states that he has seen the first trace of villi on ova half a line in size, and delineates them on an ovum of 162¾ hours, and one and a half line in size, much the same as Bischoff does. In another case Barry found none on an ovum two and a quarter lines in size. In regard to this Bischoff remarks, that he has never observed such a difference, but the smallest on which he observed the villi were, as already said, two lines in diameter. On larger ova the villi were in a corresponding degree more developed. But Barry affirms also, that he has seen in the villi from the beginning a cell-structure. This Bischoff says he can decidedly contradict, as he directed particular attention to the point.

§ 166. The ova next in degree of development Bischoff describes were elliptical, three lines in the long diameter, two and a half lines broad. Still loose in the uterus, transparent and very delicate, and requiring the uterus to be opened very cautiously for their removal. With the naked eye he remarked on the surface of the external membrane, the villi as small elevations, and on the blastodermic vesicle the *area germinativa* as an opaque point. The villi appeared under the microscope as small broad laminæ, sessile on the external membrane, and of a finely granulated texture. The blastodermic vesicle separated from the external membrane on applying to the ovum aqueous humour, and presented the same condition as the previous ova, except that the vegetative layer had already extended farther on the inner surface of the animal layer—that forming the vesicle. Both layers partake in the formation of the area germinativa (*Fruchthof.*) The two layers of the blastodermic vesicle presented the difference, that the cells of the animal layer were already more completely fused together, and more densely filled with fine molecules, and so formed a denser membrane, whilst the cells of the vegetative layer appeared still distinctly separate and very delicate and pale.

§ 167. In a stage of development close to that of the last ova, Bischoff examined others. Their presence produced a distinct dilatation of the uterus from which it was difficult to remove them uninjured in the fresh state. No intimate union however had taken place between the lining membrane of the uterus and the external membrane of the ovum, but the two surfaces were closely applied to each other, and the ova lay as if imbedded in cells produced by the dilatation of the uterus. The two ends above and below were alone free in the cavity of the uterus. The ova were three and a half to four P. lines in size, and elliptical in form. The villi were still more distinct to the naked eye—scattered in irregular groups over the whole surface of the ovum. Under the microscope they presented round notched edges, and consisted of a homogeneous transparent mass, in which dark molecules were imbedded in single groups. No cells nor nuclei to be seen. In these ova, even when quite recent, and as yet untouched by any fluid, the blastodermic vesicle no longer lay closely applied to the external membrane, but between the two there was a transparent fluid. The *area germinativa* on the blastodermic vesicle was considerably extended, but still uniformly opaque and round. The vegetative layer had become developed so much farther at the inner surface of the serous layer, that it had already extended beyond the largest diameter of the vesicle. The two layers were most intimately adherent at the *area germinativa*.

§ 168. In the next period, from about the beginning of the ninth day, it is quite impossible to take the ova uninjured out of the uterus, as the villi of their extremely attenuated and fine external membrane is in such intimate connexion with the much developed mucous membrane of that organ. In opening the uterus the external membrane is necessarily torn; the fluid between it and the blastodermic vesicle consequently escapes, and the blastodermic vesicle is found lying free in the cell of the uterus, so that this vesicle itself might be mistaken for the whole ovum, which Bischoff thinks has been done.

§ 169. Bischoff has bestowed great care to satisfy himself by observation of the actual presence at this period, of the external membrane of the ovum, which was derived from the vitellary membrane (zona), and albuminous-like investment, and to obtain by observation of the villi developed on it, the certainty that it is an important part of the future chorion. The only successful plan of opening the uterus without destroying the ovum, Bischoff found to be, to remove very carefully layer by layer the coats of the uterus on the side opposite the insertion of the mesometrium over an ovum,—the muscular, cellular, and lastly the mucous coat. But this procedure is successful in exposing the ovum whole only when *the epithelium separates from the mucous membrane, and remains adherent to the external membrane of the ovum.*

§ 170. When the ovum is thus exposed, it appears as if covered by a granular efflorescence. When Bischoff observed this appearance for the first time, he supposed that a membranous exudation had formed from the uterus around the ovum, analogous to the decidua of the human ovum. He, however, denies the existence of a decidua in the mammifera; though it is the opinion of most writers that such a structure does form. The consideration of the question does not come within the scope of the present Report.

§ 171. The mucous membrane of the uterus, Bischoff says, is at this time raised into many minute folds and villi, which are covered by sheaths of epithelium. The epithelium is no longer formed of ciliated cylinders, but of cells fused together, the nuclei of which are still quite distinct, so that a granular appearance is thereby produced. This epithelium now readily separates from the villi of the mucous membrane in a continuous layer, and remains adherent to the external membrane of the ovum, the villi of which are thrust in between the sheaths of the villi of the mucous membrane of the uterus. It presents in this form, when not highly magnified, exactly the appearance of lace, as pointed out by Coste, who called it *adventive membrane.*

§ 172. Ova exposed in the uterus in the manner above described were about half an inch in size, the blastodermic vesicle lay free within the external membrane. On that side of it turned to the insertion of the mesometrium was the *area germinativa,* still round, and either still uniformly opaque, or beginning to clear in the centre—the commencement of the central *area pellucida,* and the circumferential *area opaca,* similar to what occurs in the bird's egg. The vegetative layer of the blastodermic vesicle had extended all round, so as to form a vesicular layer within, and concentric with and closely applied to the animal layer. The clearing up of the centre of the area germinativa takes place chiefly in the animal layer.

§ 173. A few hours later the difficulty in the investigation is much increased. The blastodermic vesicle now no longer lies free, but begins to be united to the external membrane by its animal layer, and thus indirectly with the uterus. This union commences first at the side opposite the insertion of the mesometrium, and from this gradually extends to the mesenteric side. In exposing the ovum in the uterus, Bischoff proceeded as above; but now, in consequence of the alteration just described, he removed the coats of the uterus from the mesenteric side, by which however the external membrane is necessarily torn; but, as the blastodermic vesicle is not yet adherent to the external membrane at this place, it is exposed uninjured. And this place is the most important, as it is here that the area germinativa is always found.

§ 174. For examination, Bischoff cuts out a portion of the blastodermic vesicle with the *area germinativa,* and lays it on a glass plate, or in a small watchglass, in order to examine it with the microscope by transmitted light. This process is not always successful; but when it does succeed, it is seen how that the *area germinativa* not only extends farther and farther, but undergoes in quick succession changes of form. It is no longer round, but first oval and then pear-shaped, its long axis always falling on the transverse axis of the somewhat oval ovum and of the uterus. In all these forms it is composed of an opaque periphery, the *area opaca,* which incloses a clear space, *area pellucida.* These differences are owing to the different accumulation of cell-materials, especially in the animal layer; the cells appear to pass as it were from the centre towards the periphery, leaving the centre transparent. Sometimes while the *area germinativa* is still oval, but generally not until it is pear-shaped, there is seen in the long axis of the area pellucida a clear streak, at first very indistinct; with this commences the first trace of the proper embryo. This usually takes place on the eighth or ninth day post coitum.

§ 175. Although the ovum of the dog differs from that of the rabbit in some points, still the principal processes are the same in both.

In the rabbit the external membrane of the ovum, which becomes covered with villi, and by which the ovum becomes adherent to the uterus, is formed of the vitellary membrane (zona) of the ovarian ovum and the albuminous layer which is added in the Fallopian tube. In the dog, the external membrane on which villi are formed, and by which the ovum becomes adherent to the uterus, is simply, Bischoff affirms, the vitellary membrane or *zona pellucida* of the ovarian ovum without any superadded albuminous layer.

In the dog, as in the rabbit, there is formed, after a process of subdivision of the yelk and cell-formation, a blastodermic vesicle. This vesicle is composed of the two layers, animal and vegetative, which lie closely applied to each other, but yet may be separated. The commencement of the germ is a spot in the blastodermic vesicle, at first round, then elliptical, afterwards pear-shaped, in which the first trace of the embryo appears as a clear streak with opaque accumulations of matter on either side.

§ 176. Bischoff considers that there is no doubt that in these essential points the human ovum in its first development in the uterus must agree with the ovum of the mammifera. By direct observation, indeed, little or nothing is known on the point. The very early ova observed by Velpeau, by the author of this report, and by Volkmann, Bischoff is inclined to consider abnormal, and gives the following sketch of the condition he should expect the normal human ovum to present at this period: It would not lie free in the uterus, but be invested and fixed more or less in the the substance of the decidua, and that probably in the region of the orifice of one of the Fallopian tubes. It would farther be at first still like the ovarian ovum, afterwards transparent, consist of two vesicles, the outer of which, so long as the embryo had not yet appeared, would show at most the first faint traces of villi. The inner vesicle would lie more or less close to the outer, and separate from it on the application of water to the ovum. This inner vesicle would present under the microscope a cell-structure, and at one part of it either a whitish spot or a round oval or pear-shaped area germinativa must be observable. The inner vesicle would in general be very delicate, and in its already advanced stage of development would present two layers, closely adherent to each other at the embryonic spot. The size of such an ovum might be expected to be from one eighth of a line to four or five lines.

§ 177 To these statements of Bischoff it must be excepted that in ova of the latter size, the blastodermic vesicle would no longer be found filling the external membrane or chorion ; it should in fact be expected to be, relatively to the latter, small, when the small size to which the umbilical vesicle attains is considered.

The ovum, described and delineated by Dr. Allen Thomson, dating little more than fifteen days from conception, if admitted to be normal, shows how great this disproportion must be when the first trace of the embryo appears, and that the ovum described and delineated by the author of this report, before the appearance of the embryo, might not have been abnormal.

§ 178. The transparent fluid mentioned by Bischoff as at a particular stage separating the blastodermic from the external vesicle in rabbits, appears analogous to the albuminous or reticulated mass which, in the cases of Allen Thomson, and of the author of this report, filled the greater part of the interior of the external membrane or chorion.

### V. Amnion, chorion, umbilical vesicle, and allantois.

§ 179. In 1828 there appeared in the second volume of Burdach's Physiology a pretty complete history of the ovum of the mammifera, from the first appearance of the embryo to the development of all its important parts. The materials for this history were derived from the labours of Prevost and Dumas, Bojanus, Baer, to which may be added those of Oken, Kieser, Meckel, Rathke, J. Müller, and others.

§ 180. A complete continuous series of observations however was wanting. Coste made a step towards supplying this desideratum by tracing the development of the ovum in the sheep, dog, and rabbit. Though in these researches he confirmed much of what had been previously done in Germany, he did not establish anything new of importance.

§ 181. In the second volume of Baer's 'Entwickelungs-geschichte,' published in the same year (1837) with Coste's 'Embryogenie comparée,' there is brought together the history of the development of the embryo and ovum in almost all orders of mammifera and in man, and a perfect analogy is shown to exist in the development of the embryo, not only among the different mammifera but between them and birds. The completeness of this analogy, which had lately been called in question, Bischoff has established beyond all cavil.

§ 182. The first processes in the development of the embryo go on very rapidly, seeing that in the rabbit, from the appearance of its first trace to that of almost all the important organs scarcely two days elapse,—the ninth and tenth days.

§ 183. As was above seen, after the area germinativa comes to present a central pellucid and a peripheral opaque part, and passes from the round to the pear shape; a bright streak appears on the long axis of the pellucid part. Close around this streak, but especially on either side of it, formative matter collects and constitutes a somewhat opaque plate of the same form as the area germinativa itself, consequently oval or pear-shaped. It is principally in the animal layer that these changes occur. The bright streak is a groove in that layer which is extremely thin, and therefore quite transparent at the place. Viewed from above, the two edges of this groove at the end corresponding to the broad part of the pear-shaped area germinativa, pass into each other by a small arch, whilst at the other end they unite in a point. The former is the head, the latter the tail end of the embryo about to be formed.

§ 184. The accumulations of formative matter on either side of the groove exist likewise only in the animal layer. The subjacent vegetative or mucous layer is almost uniformly opaque in the whole extent of the area germinativa by which the distinction between the pellucid and opaque divisions which the animal or serous layer presents at that place is greatly lessened. Corresponding to the bright groove in the pellucid part of the animal layer, there is in the vegetative layer a faint clear streak, which, however, appears to be merely an impression made by the groove of the animal layer. At the groove the two layers adhere so closely that it seldom happens that a separation is effected between them at this place without laceration.

§ 185. The groove in question is what has been called the primitive streak, and was considered by Baer and others as an actual linear accumulation of matter. This, however, was shown not to be the case in the bird's egg, by Coste and Delpech, and more recently by Reichert. The observations by Bischoff on the mammiferous ovum, just detailed, show that in it also the primitive streak is simply a groove. The same thing is agreeable to Reichert's and the author's own observations on the batrachian reptiles. Instead therefore of *primitive streak*, Bischoff employs the expression *primitive groove.*

§ 186. The two accumulations of formative matter on either side of the groove, Bischoff does not with Reichert consider to be the original halves of the nervous system, but the *original basis of the body of the embryo.*

§ 187. The *area opaca* having much extended itself and again become round, and the *area pellucida* and the basis of the body of the embryo having become fiddle-shaped, that portion of the formative matter which immediately constitutes the two sides of the primitive groove, rises up in the form of "dorsal plates." These meeting in the middle over the groove convert it into the canal in which the central organs of the nervous system are formed. Subsequently the circumferential part of the same formative matter becoming thickened, and bending downwards and inwards constitutes "visceral plates." The area pellucida is now reduced to a crescentic patch around the head end of the embryo.

§ 188. From this it is seen that the first development of the embryo of the mammifera is analogous to that of the embryo of the bird. The farther development of the embryo and its organs is so likewise. It is not, however, purposed here to follow the development of the embryo itself further in detail. Let it suffice to observe, that the dorsal plates having closed in, the formation of the nervous centres commenced, and the head end of the dorsal canal become dilated, the embryo bends forward on itself, and an elevation of its anterior, and in a less degree of its posterior ends takes place from and above the plane of the blastodermic vesicle, so that the embryo is, as it were, constricted off from it The sides still pass gradually into the blastodermic vesicle; but by and by the same thing takes place here also. This change is owing to the margins of the visceral plates all round, approaching each other below. A cavity is thus inclosed, named the visceral cavity, communicating with that of the blastodermic vesicle at the place of constriction, between it and the embryo.

§ 189. It is to be particularly remarked that the whole embryo is as yet composed only of the thickened central part of the animal layer; the vegetative layer lies quite smooth at its under surface. The vegetative layer, however, takes part in the constriction which occurs between the embryo and the blastodermic vesicle. Above and below it is drawn within the visceral tube, which is in the process of formation. If the embryo be now examined from the ventral side, the head and tail ends are seen covered by the vegetative layer from the place to which the constriction has extended, from the place of entrance therefore into the upper and lower part of the visceral tube. Those parts of the vegetative layer covering the head and tail ends of the embryo have been called *involucra*—of the head and of the tail.

§ 190. In the formation of the head and tail involucra, the animal layer does not take part in the same manner as the vegetative, in consequence of a change in the former now taking place, which gives origin to a new formation and a change in all the relations of the ovum.

§ 191. If at this time the head end of the embryo, (at the tail end the formation is still too little advanced,) be examined with the greatest care under a magnifier, and with fine needles, it is found that it does not lie free on the blastodermic vesicle, but is covered by a double fold of an extremely fine and transparent membrane. This membrane is a part of the animal layer of the blastodermic vesicle undergoing a metamorphosis towards the formation of the amnion.

§ 192. The fold of the animal layer extends more and more over the head end, in an arched line towards the middle of the back. While this is going on, the same process takes place at the tail end; and soon after from the lateral margins of the embryo also. In this manner the animal layer reflects itself from the whole periphery of the body of the embryo, which is merely its developed central part, over the back of the embryo, and then abruptly folding on itself, extends back peripherally. From all sides the folds approach more and more the middle of the back, until at last the margins of the folds meet together in a point. The inner layer of the fold thus comes to cover the whole body of the embryo, lying so close on it, and being so fine that without particular manipulation it is not at all to be recognized. The outer or upper layer again forms a continuous membrane. Only at the point of closure of the folds, the two layers continue for some time united, but eventually separate from each other entirely. The layer lying close on the embryo, and inclosing it is the *amnion;* the outer layer is still the animal, but is now called *serous* investment, (false amnion of Pander in the bird,) and lies, so far as it is entirely separated from the inner, close to the outer membrane of the ovum, with which Bischoff affirms it coalesces entirely. It is now by this coalescence of the serous investment with the outer membrane of the ovum that the formation of the *chorion* is, according to Bischoff, completed.

§ 193. The above-described mode of development of the amnion, first discovered in the bird, has been observed and traced by Baer in the sheep, sow, and dog.

§ 194. From his observations on the ova of the cat, sheep, and rabbit, Dr. A. Thomson is satisfied that the amnion is formed by the union of the cephalic and caudal folds of the serous layer of the germinal membrane in the mammalia, nearly if not exactly in the same way as was shown to take place in birds by Pander. In the animals mentioned he has examined the amnion in its open state before the union of its cephalic and caudal folds.

§ 195. Coste gives a different view of the formation of the amnion in the mammifera. He supposes that it is first formed by the detachment or rising of the outer covering or epidermis of the fetus.

§ 196. Dr. Barry, while he adopts the explanation which has been given of the manner of formation of the amnion in the bird, says he must be understood as maintaining, in opposition to the view of others, that the membrane so appropriated in mammalia, is no part of that structure out of which the embryo is formed. The membrane he refers to as forming the amnion, is that which was above spoken of as the blastodermic vesicle of Bischoff, the animal and vegetative layers inclusive.

§ 197. The gelatinous-looking investment which, as above seen, the ovum in the Fallopian tube presents in addition to the vitellary membrane (zona) of the ovarian ovum, was first indicated by the author of this report as the future chorion. Dr. Barry, when he had become acquainted with the structure in his 2d series, and had in consequence renounced all he had said in his 1st series, (p. 316,) under the head of "The true chorion, a structure superadded within the ovary in the class mammalia," so far confirmed what had been indicated by the author of this report, that he traced the structure up to the period when villi formed on it in the uterus.

§ 198. The author of this report having observed that at a certain stage the vitellary membrane (zona) is no longer visible, supposed that in the progress of development it gives way, and the gelatinous-looking investment comes to constitute alone the chorion. Barry maintains that the vitellary membrane (zona), (not his vitellary membrane), on the contrary continues distinct. Bischoff admits that the vitellary membrane does cease to be distinct from the gelatinous-looking investment, but maintains that it does not give way and dissolve, but coalesces as above seen with the gelatinous-looking investment.

§ 199. Bischoff says, (p. 118,) "I believe I have thus proved that the amnion in the mammifera is a product of the development of the peripheral part of the animal layer of the blastodermic vesicle, and that the chorion is a very complicated structure formed, in the rabbit, of the vitellary membrane (zona) of the ovarian ovum, of the albuminous layer added in the passage of the ova through the Fallopian tubes, and likewise of the peripheral part of the animal layer of the blastodermic vesicle, which on its union with the chorion was called serous investment."

§ 200. In the dog's ovum, around which, as above seen, he affirms that no albuminous layer is formed in passing through the Fallopian tube, Bischoff says the vitellary membrane (zona) is itself the part on which the villi form. But Bischoff mentions (p. 119) his having observed appearances in the rabbit which might be taken to indicate that the external membrane of the ovum composed of the coalesced vitellary membrane (zona) and albuminous investment dissolves at a certain stage, and that then the serous investment derived from the animal layer of the blastodermic vesicle comes alone to form the chorion. The chorion is thus, according to Bischoff, either a combination of the external membrane of the ovum resulting from the coalescence of the albuminous layer and vitellary membrane (zona) and of the serous investment; or the chorion consists of the serous investment alone.

§ 201. Reichert* doubts the coalescence of the vitellary membrane (zona) with the gelatinous-looking investment, and is inclined rather to the opinion that the vitellary membrane sooner or later disappears. In regard to Bischoff's statement that the chorion is always a product of development from the ovum, and not derived from the maternal organism, Reichert, while he justly admits the correctness of it, observes that the proposition is not in agreement with what Bischoff says regarding the origin of the gelatinous-looking investment. (See § 85.)

§ 202. About the time the amnion begins to be formed, the vascular system first appears in the form of a *heart-canal,* in the anterior wall of the head end of the embryo, where it is continued into the blastodermic vesicle, of a *vena terminalis* at the periphery of the area opaca, and, throughout the latter now becoming area vasculosa, of slight traces of a *vascular network* between the vena terminalis and the under crura of the heart-canal. The vessels in the area vasculosa lie between the animal and vegetative layers, and constitute, according to Bischoff, a distinctly demonstrable *vascular layer.* He has quite certainly separated with the needle this vascular layer from the outer surface of the vegetative layer as an independent membraneous formation, as far as the circumference of the body of the embryo. (p. 122.)

§ 203. The amnion having been formed, the development of the intestinal canal goes on; and during this the embryo, by development and approximation of the visceral plates, becomes more and more separated by constriction from the vegeta-

* Beiträge zur Kenntniss des Zustandes der heutigen Entwickelungs-geschichte.—Berlin, 1843, p. 8.

tive and vascular layers of the blastodermic vesicle, which now constitutes the *umbilical vesicle*. The vessels of the vascular layer, consisting of one or two veins carrying blood to the embryo and an artery proceeding from it, are the omphalo-mesenteric. The canal to which the communication between the intestine and umbilical vesicle is now reduced is the *ductus omphalo-mesentericus* or *vitello-intestinalis*.

§ 204. Bischoff observes that this mode of formation of the umbilical vesicle is the same in all the mammifera. In regard to the open communication between the umbilical vesicle and intestine, which has been so much contested, there can be no doubt. He has examined numerous embryos of the dog, cow, rabbit, and rat, in which this communication was as evident to the senses as it must be to the mind after some knowledge of the whole process of development. In the further relations of the umbilical vesicle to the embryo and the ovum there take place in the different orders of the mammifera great differences.

§ 205. Whilst the mucous and vascular layers of the blastodermic vesicle begin to be separated by constriction from the embryo in the form of umbilical vesicle, there is observed sprouting out at the lower end of the embryo, already become free by constriction, a small very vascular vesicle which is at first round, afterwards pear-shaped. This production is the *allantois*, a part of the greatest importance in the further development of the embryo and ovum.

§ 206. It has generally been supposed, with Baer, that the allantois is first developed as a hollow diverticulum from the lower end of the intestine, and consists like it of a vascular and mucous layer. Recently it has been affirmed by Reichert that in the bird the allantois presents itself originally in the form of two small solid prominences at the end of the Wolffian bodies, and in connexion with their excretory ducts; but he does not derive the allantois from the Wolffian bodies. These prominences gradually coalesce and form at first a flattened elevation, which is soon converted into a vesicle. Coste again describes the allantois as an immediate production from the blastodermic vesicle. Bischoff, although he does not agree with Coste's views, admits his observation to be correct. From his researches on the rabbit, Bischoff confirms Coste's statement that the first appearance of the allantois takes place before the intestine is formed, and also before there is even a trace of the Wolffian bodies. In the embryos of rabbits in which the mucous and vascular layers of the blastodermic vesicle were still evenly continuous with the two sides of the embryo, and only the head and tail end, the latter still quite round, were separated from it by constriction, Bischoff has seen the first trace of the allantois at the tail end, in a form such as is represented by Coste. There was no appearance of the end part of the intestine, which is already formed in embryos a little older; and of the Wolffian bodies Bischoff could not discover any trace under the microscope. The basis of the allantois appeared to him as a growth from the visceral plate of the tail,—as a mass of cells not yet hollow, but in which there already existed a great number of vessels. Afterwards, when the allantois has become a vesicle, it is truly in connexion with the intestine and the excretory duct of the Wolffian body; but how this comes about Bischoff has not yet been able to determine.

§ 207. When the allantois has distinctly become a vesicle, it and its vessels grow quickly. The vessels are the future umbilical vessels. By the closing in of the visceral plates, in the formation of the abdominal walls and navel, the vesicle represented by the allantois is divided by a constriction into two compartments. That which is within the embryo is the urinary bladder; that which is without is the allantois, properly so called, and is disposed differently in different animals. The canal at the constricted place, and which afterwards becomes a mere cord, is the *urachus*.

§ 208. The peripheral part of the allantois soon forms, as is known, one of the most important parts of the ovum. Hitherto the ovum had received what was required for its development and nourishment, merely by imbibition through its external membrane, but now the important business of drawing nourishment from the mother is transferred to the vessels of the allantois. The allantois with its vessels turning to the right side of the embryo, and at the same time twisting the lower end of the latter from left to right, grows rapidly towards the chorion, applies itself and becomes adherent to it. The allantoidal vessels not only pass into the chorion but are prolonged into the villi which here undergo a remarkable degree of development. At the corresponding place the mucous membrane and vascular system of the uterus in like manner become unusually developed. The two vascular systems, that of the allantois in the villi of the chorion on the one hand, and that of the uterus on the other, though not in direct communication come to be in very close connexion. There is thus formed the *placenta.*

§ 209. The development of the allantois and its vessels, like that of the umbilical vesicle is very different in the different orders of mammifera.

§ 210. Having traced the origin of the amnion, chorion, umbilical vesicle, and allantois in the rabbit, a brief statement may now be made of what is known, or supposed to be known on the subject in man. The closest correspondence, it will be observed, exists between the human ovum and that of the other mammifera.

§ 211. *Amnion.* From their observations Professors Baer and A Thomson hold, that in the human ovum the amnion is formed in the same way as in the mammifera and birds, only it is to be remarked that the ununited state of the cephalic and caudal folds has never yet been observed. In the very early human ovum described by Allen Thomson, he did not detect an amnion. He says the embryo was fixed by its back to the chorion. This Bischoff interprets as follows: The folds of the serous layer which forms the amnion, had closed over the back of the embryo and the outer layer, the serous investment had applied itself to the chorion, whereby the embryo appeared fixed to the chorion by means of the amnion at the place of closure of the folds. From what he has observed in the mammifera, Bischoff thinks this union is afterwards dissolved, but the author of this report believes that in man the union at the point of closure never dissolves. In the human ovum the amnion is always adherent at one point to the chorion, and that appears to be the place where the amnion closed or tended to close in. It is the place also near which the allantois joins the chorion.

§ 212. *Chorion.* It has been seen that Bischoff is of opinion that the human ovum does not in its passage along the Fallopian tube become covered by an albuminous investment, but that the external membrane in the uterus is the vitellary membrane (zona) of the ovarian ovum. The chorion, he believes, results from this vitellary membrane (zona), combined with the serous investment of the blastodermic vesicle. That this is the case the author of this report is much inclined to doubt. The latter part of the statement in particular he denies; for if the early ovum described by Dr. Allen Thomson be admitted to have been normal, the blastodermic vesicle is too small for its serous layer to have yielded a serous investment to the chorion, and that after forming the amnion. From an observation which the author of this report himself made not long ago, he is inclined to believe, as was originally suggested by Baer, and observed by A. Thomson, that the serous lamina of the blastodermic vesicle in the human ovum does not go to form a serous investment to the chorion, between which and it there is so large a space filled with a gelatiniform substance; but after forming the amnion, the rest of it remains adhering as a very delicate film to the blastodermic, now umbilical vesicle.

§ 213. The chorion has originally no vessels of its own. It is only where it has been joined by the allantois that its villi contain vessels, and that only at the place where the placenta forms. In pachydermata, ruminants, and carnivora, the allantois being extensively developed, lines the whole of the chorion, which is thus found in the form of a vascular membrane, as indeed is also the amnion from the same cause.

§ 214. *Umbilical vesicle.* In regard to this there is a great difference between the human ovum and that of most of the mammifera; for whilst in the latter the umbilical vesicle attains a very large size, and remains distinct throughout the whole of fetal life, and even in some, as the rodents, plays an important part by carrying vessels to the whole of the chorion with the exception of that part which corresponds to the placenta; in man it attains a very slight degree of development, quickly loses its importance for the embryo and ovum, and sooner or later wholly disappears. The great length into which the omphalo-mesenteric duct, now become closed, is drawn in the human ovum, is a peculiarity connected with another, viz., the length of the umbilical cord. The cases observed by Allen Thomson, Coste, Wagner, Müller, the author of this report, and others, remove all the doubt which was originally entertained as to the omphalo-mesenteric duct being really a free communication between the intestine of the embryo and the umbilical vesicle.

§ 215. *Allantois.* In human ova between the third and fourth week, described by Wagner, Müller, Coste, Allen Thomson, Meckel, and Baer, the allantois presented itself as in the mammifera in the form of a small elongated vesicle, which protruded from the lower end of the embryo, and applied itself by its broad base to the chorion. In later ova this vesicle is no longer found, but there is in its stead a cord extending from the embryo to the chorion containing the umbilical vessels. The observation of ova after the disappearance of the allantois formerly led to the erroneous supposition that either no allantois exists in the human ovum, or that it quickly attains a great degree of development, and adheres by one layer to the inside of the chorion, and by another to the outside of the amnion as in the pachydermata and ruminants. Adopting this latter view, Velpeau maintained that the gelatiniform substance or *magma reticulé*, between the chorion and amnion is the contents of the allantois, but to say nothing of the erroneousness of the view that the allantois attains such a great degree of development, the observations of Dr. Allen Thomson, and of the author of this report, show that the *magma reticulé* exists before the allantois itself appears.

### POSTSCRIPT.

§ 216. Since the preceding report was printed, the author has seen in the "Comptes rendus hebdomadaires" of the French Academy of Sciences for July 17th, 1843, an extract of a letter from Professor Bischoff to Professor Breschet, on the detachment and fecundation of the ova of man and the mammifera. In this communication Bischoff expresses himself as being now satisfied that the detachment of the ova from the ovaries does not, as he formerly supposed, take place only after coitus, nor of course depend on the direct contact of the seminal fluid with the ovary, but that ova come to maturity at more or less regular periods, and are spontaneously detached from the ovaries whether coitus take place or not; and consequently that in some cases it must happen that ova are detached before, and in other cases not till after coitus. Under ordinary circumstances, however, the detachment occurs after coitus; for, according to Bischoff, it is during the maturation of the ova in the ovaries that animals enter into heat, and are thus impelled to coitus before detachment takes place.

§ 217. The following case Bischoff adduces to show that ova may be detached from the ovaries before coitus. A young and strong bitch which had never pupped was watched at the time of its coming into heat, and after being in this state several days, was lined. Immediately after coitus, which lasted a quarter of an hour, Bischoff extirpated the left horn of the uterus along with the Fallopian tube and ovary of the same side. On examination with the microscope, semen was found to have penetrated as far as the superior angle of the horn of the uterus and the spermatozoa moved actively. No trace of semen was observed in the tube, but the ovary already presented burst Graafian follicles and distinct *corpora lutea.* In the Fallopian tube *five ova* were discovered, already advanced fifty-five millimeters from its abdominal orifice.

The animal was killed twenty hours after the coitus, and on examination of the parts of the right side, spermatozoa, still in motion, were found, not only towards the horn of the uterus, but advanced six millimeters in the Fallopian tube. There were five *corpora lutea* in the ovary, and *five ova* in the middle of the tube. No spermatozoa were observed around the ova, which Bischoff thinks was owing to the ova on the one hand, and semen on the other not having met The ova, therefore, could not yet have been fecundated.

§ 218. Admitting, as is already done in the preceding report, that ova come to maturity at more or less regular periods, and are spontaneously detached from the ovaries, the question arises, Are such ova as are detached before coitus capable of being fecundated should coitus take place within a certain time after? Bischoff answers this in the affirmative, but has not yet brought forward facts to prove that it is so. The author of this report must confess that he did not contemplate the likelihood of the occurrence when he maintained the proposition that it is not until the ova have been detached from the ovaries and received into the Fallopian tubes, that fecundating contact takes place between them and the semen,—a proposition which it will be seen Bischoff also now adheres to. And though now that the question has been brought before him, the author of this report does not venture to deny the probability of the occurrence, he must observe that there appears to him a difficulty standing in the way in regard to *corpora lutea*, at least in the human subject. Were "corpora lutea" always formed in the ovaries after the detachment of ova, whether coitus has taken place or not, they ought to be found in the ovaries of every woman at the child-bearing time of life. Hitherto, however, the result of observation has been, that they occur occasionally only, and that mostly in cases in which there has recently been conception. The occurrence of yellow bodies in the ovaries, without previous conception, is not so common, and then there is reason to believe that they are different in their structure from those occurring after conception.

§ 219. Supposing, however, with Bischoff, that there is no difference in the *corpora lutea* formed under these two different circumstances, it would be difficult to account for their all but invariable occurrence after conception, and their occasional occurrence only independent of it. But if there is, as the author of this report believes, an actual difference in the *corpora lutea* formed under these two different circumstances, it must be admitted that the peculiarities of those corpora lutea which occur after conception are connected with some peculiarity in the process of detachment from the ovaries of ova destined to be fecundated; and such peculiarity, it is to be remarked, can only be determined in some way by the coitus, from which the conception results. If this be so, it would follow that the detachment from the ovaries of ova destined to be fecundated, must be subsequent to coitus. But the point is one on which much light may be thrown by farther observation.

